# Low impact of polyploidization on the transcriptome of synthetic allohexaploid wheat

**DOI:** 10.1186/s12864-023-09324-2

**Published:** 2023-05-11

**Authors:** Meriem Banouh, David Armisen, Annaig Bouguennec, Cécile Huneau, Mamadou Dia Sow, Caroline Pont, Jérôme Salse, Peter Civáň

**Affiliations:** 1 INRAE/UCA UMR 1095, 5 Chemin de Beaulieu, Clermont Ferrand, 63100 France; 2 Institut de Génomique Fonctionnelle de Lyon, CNRS UMR 5242, 46 allée d’Italie, Lyon, 69364 France

**Keywords:** RNA-seq, Whole-genome duplication, Genomic shock, Transposable elements, Leaves, Grain

## Abstract

**Background:**

Bread wheat is a recent allohexaploid (genomic constitution AABBDD) that emerged through a hybridization between tetraploid *Triticum turgidum* (AABB) and diploid *Aegilops tauschii* (DD) less than 10,000 years ago. The hexaploidization can be re-created artificially, producing synthetic wheat that has been used to study immediate genomic responses to polyploidization. The scale of the consequences of polyploidization, and their mechanism of establishment, remain uncertain.

**Results:**

Here we sampled several synthetic wheats from alternative parental genotypes and reciprocal crosses, and examined transcriptomes from two different tissues and successive generations. We did not detect any massive reprogramming in gene expression, with only around 1% of expressed genes showing significant differences compared to their lower-ploidy parents. Most of this differential expression is located on the D subgenome, without consistency in the direction of the expression change. Homoeolog expression bias in synthetic wheat is similar to the pattern observed in the parents. Both differential expression and homoeolog bias are tissue-specific. While up to three families of transposable elements became upregulated in wheat synthetics, their position and distance are not significantly associated with expression changes in proximal genes.

**Discussion:**

While only a few genes change their expression pattern after polyploidization, they can be involved in agronomically important pathways. Alternative parental combinations can lead to opposite changes on the same subset of D-located genes, which is relevant for harnessing new diversity in wheat breeding. Tissue specificity of the polyploidization-triggered expression changes indicates the remodelling of transcriptomes in synthetic wheat is plastic and likely caused by regulome interactions rather than permanent changes. We discuss the pitfalls of transcriptomic comparisons across ploidy levels that can inflate the de-regulation signal.

**Conclusions:**

Transcriptomic response to polyploidization in synthetic AABBDD wheat is modest and much lower than some previous estimates. Homoeolog expression bias in wheat allohexaploids is mostly attributed to parental legacy, with polyploidy having a mild balancing effect.

**Supplementary Information:**

The online version contains supplementary material available at 10.1186/s12864-023-09324-2.

## Background

Bread wheat (*Triticum aestivum* L.) is one of the world’s crucial staple crops [1, 2]. Considerable efforts are being dedicated to understand its genetic organization, diversity and environmental adaptations, paving the way towards improved performance through genome-informed breeding. Bread wheat is an allohexaploid (2n = 6x = 42 chromosomes; genomic constitution AABBDD) that emerged through a hybridization between allotetraploid wheat *T. turgidum* (AABB) and *Aegilops tauschii* (DD), and subsequent whole-genome duplication [3]. Since the donor of the AABB genomes appears to be a free-threshing, durum-like wheat [4, 5] and the major contributor of the D genome is currently distributed along the south-western shores of the Caspian Sea [6], wheat allohexaploidization likely occurred after the domestication and spread of free-threshing tetraploids, i.e. less than 10,000 years ago.

Thanks to the allohexaploid nature of bread wheat, most genes have corresponding copies (homoeologs) originating from the three parental subgenomes. This pool of variably diverged gene variants is assumed to provide a heterosis effect that could be behind the crop’s increased yield (compared to einkorn and emmer [7]) and adaptive plasticity [8, 9]. However, transcriptomic studies across several tissues and developmental stages in wheat [10, 11] have revealed that 21.1-37.4% of triads (sets of three homoeologous genes, one on each subgenome) have one or two gene copies silenced. This homoeolog expression bias, i.e. unequal contribution of homoeologous genes to the total expression of a triad, is usually consistent across different tissues [10], meaning that many genes appear to be permanently silenced in allohexaploid wheat.

The above observation seemingly fits the notion of a ‘genomic shock’, usually denoting massive genetic and epigenetic changes that manifest immediately (or within a few generations) after polyploidization [12]. The genomic shock includes widespread reprogramming of gene expression, activation of transposable elements (TEs), structural rearrangements, homoeologous exchange and epigenetics [13, 14], either of which has been reported in various synthetic allopolyploids (i.e. allohexaploids created experimentally, using wide crosses and induction of whole-genome duplication). In nascent wheat synthetics created from *T. turgidum* and *Aegilops* sp., Ozkan et al. observed directional and reproducible sequence elimination, and an overall reduction in genome size by 4–8% occurring at the hybrid stage [15, 16]. Gene loss was also detected in synthetic wheat allotetraploids [17], however, no sequence elimination was observed by Mestiri et al. [18], although they only studied homoeologous group 3 chromosomes. Dynamic changes were observed in TEs, including massive deletions and retrotransposition bursts [19], frequent changes in the methylation status of several TE families [19, 20], and transcriptional activation of the *Wis 2-1 A* retrotransposon associated with silencing or activation of adjacent genes [21]. A possible link between TEs and gene repression was also suggested [22], based on increased density of small interfering RNAs (siRNAs) at TE-associated D-homoeologs of nascent wheat allohexaploids, which may account for their biased repression. Furthermore, different epigenetic responses (siRNA, chromatin modifications) to polyploidization were reported for SSAA (*Ae. speltoides × T. urartu*) and AADD (*T. urartu × Ae. tauschii*) synthetics [23].

Gene expression changes are among the most studied consequences of polyploidization, and the ‘transcriptomic shock’ has been repeatedly assessed in wheat synthetics. Prior to the availability of an annotated reference genome, changes in gene expression have been evaluated in microarray experiments, which were unable to differentiate individual homoeologous copies and could not therefore detect homoeolog expression bias or differential expression (DE) of individual genes. Nonetheless, the microarray studies were able to compare the total expression of a ‘gene’ (across its homoeologs and possibly also paralogs) in a synthetic to the midparent value (MPV) obtained from the parents (either by calculation or mixing of the parental RNA samples). Pumphrey et al. [24] reported that ~ 16% of wheat ‘genes’ are expressed non-additively in allohexaploid synthetics, i.e. differ significantly from MPV. Akhunova et al. [25] have detected an even higher proportion of non-additively expressed genes (19%) in synthetic allohexaploids; while 30.7–56.5% of ‘genes’ were found to be non-additive in the leaf and young inflorescence of allotetraploids synthesized from *T. urartu* and *Ae. longissima* [26]. Similarly high levels of non-additive expression were observed in transcriptomes of SSAA and AADD synthetics (35% and 20%, respectively) [23]. However, other microarray-based studies concluded that the vast majority of ‘genes’ in genetically stable allohexaploid synthetics is expressed additively, with only 0.7-7% ‘genes’ differing from MPV [27, 28].

Relatively few studies have taken advantage of the transcriptomic approach to examine the homoeolog expression bias in nascent wheat allohexaploids. Hao et al. [29] reported that the D-subgenome is massively affected by the transcriptomic shock, with downregulation being the dominant change (32.6% of D-homoeologs down-regulated), while changes on A and B are infrequent. It was also established that the transcriptomic shock is not caused by the whole-genome duplication per se, but rather occurs at the allotriploid stage after the interploidy cross between *T. turgidum* and *Ae. tauschii*, and the transcriptional remodelling is partially reversed in the first allohexaploid generation [29]. Ramirez-Gonzales et al. [10] have analysed this dataset further and found no relationship between the presence of TEs in promoter regions and altered expression patterns between homoeologs in dominant and suppressed triads.

The above overview of the published literature demonstrates little consistency of results regarding the consequences of the genomic shock in nascent wheat allopolyploids. This lack of consistency pertains to the occurrence of sequence elimination, involvement of TEs in de-regulation of nearby genes, the extent of the transcriptomic remodelling, as well as the dominant pattern of gene expression changes. Due to the conflicting results, but also a lack of comprehensively sampled data, questions persist regarding the role of the parental genotypes, maternal effects, stochasticity and heritability of the changes induced in nascent polyploids, and ultimately, about the underlying molecular mechanisms of these changes and their relevance for the plasticity of bread wheat.

Here, we present a comprehensive transcriptomic analysis of several allohexaploid synthetics derived from two alternative AABB parents and two alternative genotypes of *Ae. tauschii*. Our comparisons include different tissues (mature leaf and developing grain), consecutive generations, and synthetics derived from reciprocal crosses. First, we quantified DE between the parents and the synthetics considering all genes (not limiting the analysis to triads) and TE families. Additionally, we analysed homoeolog expression bias and the relationship between DE, homoeolog bias and TEs. We identified potential sources of systematic bias arising from the difficulties of inter-ploidy comparisons involving recently-diverged species, and we describe our mitigation strategy in the Supplementary Note (Additional File 1). Finally, we provide an updated and over-arching view on the extent of transcriptomic changes in wheat synthetics and discuss their relevance for wheat breeding.

## Results

### Descriptive statistics of the transcriptomes

In the present study, we analysed leaf and grain transcriptomes in eight parent-synthetic combinations that are based on two different genotypes of *T. turgidum* subsp. *durum* (Langdon and Joyau) and two different genotypes of *Ae. tauschii* (*Ae. tauschii*-87 and *Ae. tauschii*-109). The synthetics (Table 1) are named according to their parental combination (e.g., Lx109, indicating a cross Langdon × *Ae. tauschii*-109), with the polyploid generation appended at the end (C1-C4 or S5; see Methods).

**Table 1:** Basic characteristics of the synthetic wheat samples analysed in this study

synthetic allohexaploid wheat	maternal parent (genome constitution)	paternal parent (genome constitution)	tissue analysed	generation since polyploidization
109×L-C1	*Ae. tauschii-*109 (DD)	Langdon (AABB)	leaf	1
109×L-C2	*Ae. tauschii-*109 (DD)	Langdon (AABB)	grain	2
109×L-C3	*Ae. tauschii-*109 (DD)	Langdon (AABB)	leaf	3
109×L-C4	*Ae. tauschii-*109 (DD)	Langdon (AABB)	grain	4
Lx109-C1	Langdon (AABB)	*Ae. tauschii-*109 (DD)	leaf	1
Lx109-C2	Langdon (AABB)	*Ae. tauschii-*109 (DD)	grain	2
J×109-S5	Joyau (AABB)	*Ae. tauschii-*109 (DD)	grain	5
J×87-S5	Joyau (AABB)	*Ae. tauschii-*87 (DD)	grain	5

**Fig. 1:**
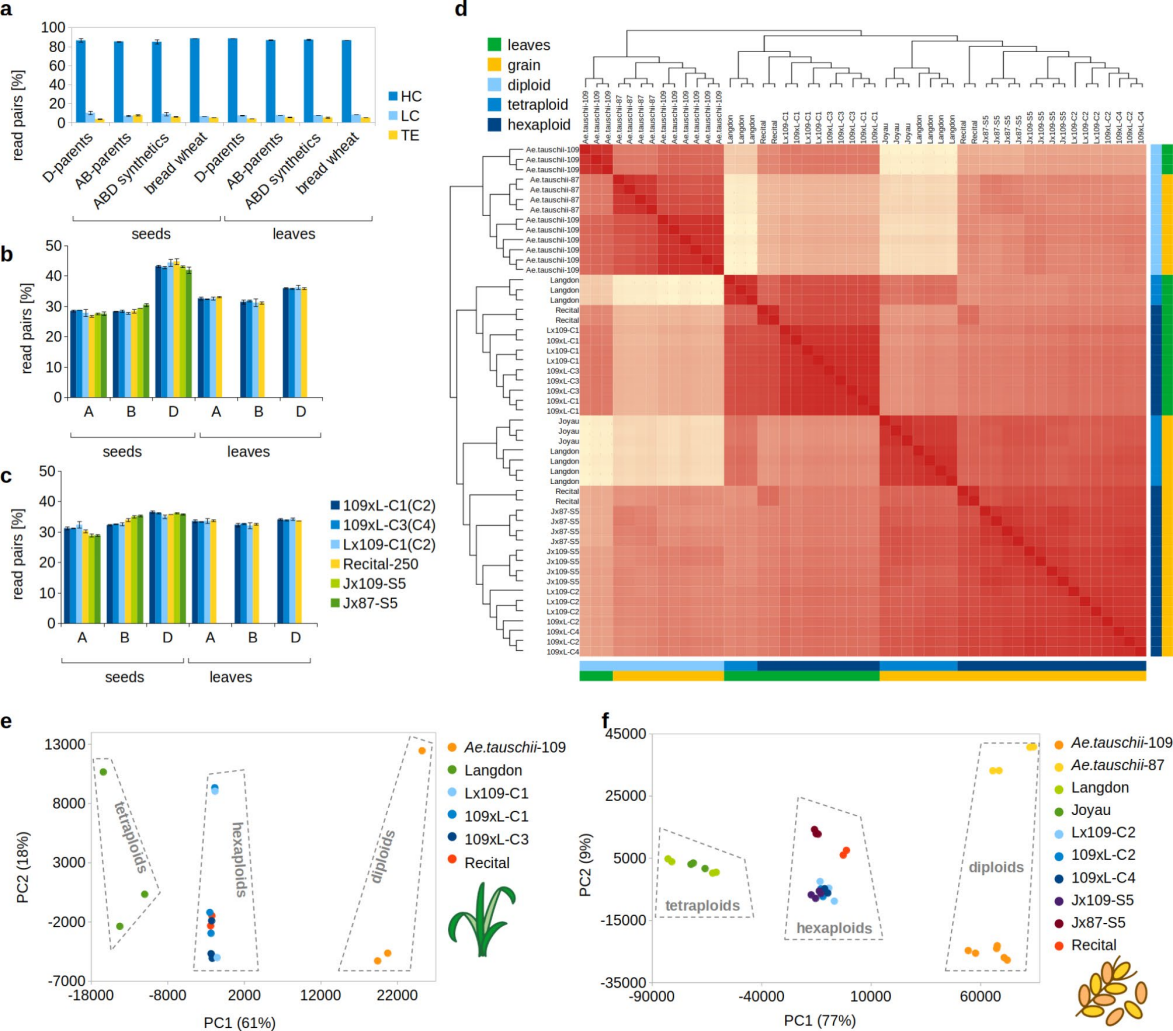
Descriptive statistics and integrity of the RNA-Seq libraries. **a**: Proportions of read pairs mapped to high-, low-confidence genes and TEs. The graph shows means of replicates, with error bars indicating standard deviation. **b**: Proportions of read pairs mapped to A, B and D subgenomes of the allohexaploid genomes. The graph shows means of replicates, with error bars indicating standard deviation for the STAR-pipeline. **c**: the same as b, but for the BOWTIE2-pipeline. **d**: A heatmap of all transcriptomes (BOWTIE2-pipeline). **e**: Top two principal components of a PCA for the leaf transcriptomes (STAR-pipeline). **f**: The same as e, but for the grain transcriptomes

We used Illumina technology to obtain between 66 and 124 million read pairs (2 × 150 bp) per RNA-Seq library, totalling 1.37 Tb of data (Supplementary Tables 1, Additional file 2). The read mapping and summarization, together with the downstream analyses, were performed with two different pipelines in parallel (see Methods and Supplementary Tables 2, Additional file 2). In the Bowtie2-pipeline that allows to analyse TE transcription, 9% of the entire set of raw reads were discarded during the quality trimming and reference mapping steps, and 87.7% of the retained reads were assigned to annotated features. Most of the RNA-seq read pairs were assigned to high-confidence genes (86.1%; mean of all libraries), followed by low-confidence genes (8.5%) and TEs (5.4%) (Fig. 1a). Differences in TE-derived read proportions between natural (Recital) and synthetic allohexaploids were mostly insignificant, except for Jx87-S5, Jx109-S5 and 109xL-C4 (all grains), where the TE-derived reads were slightly higher. We observed a strong and significant difference between the proportion of TE-derived reads of *Ae. tauschii* (3.7% of the total reads, averaged across all libraries) and durum wheat (7.2%). In line with expectations, the proportion of TE-derived reads in the synthetics is in between these values (5.6%).

For allohexaploids, we summarized the overall contribution of the A, B and D subgenomes to the total transcription by summing up RPMs (Reads Per Million) of subgenome-assigned genes. In the STAR-pipeline, the D-subgenome contribution is higher than both A and B contributions in both tissues (Fig. 1b), suggesting that D transcription dominates over the other two subgenomes. However, this could be due to systematic differences in gene annotations (e.g. different stringency for calling high-confidence genes) in the *Ae. tauschii* v4.0 and Svevo v2 reference genomes that were used in this pipeline. In the Bowtie2-pipeline, where the Chinese Spring genome was used, the difference between D and the other two subgenomes is less obvious, though still significant (T-test p-value < 0.05 in all synthetics, except for A-D in the leaves of Lx109-C1 and 109xL-C1; Fig. 1c), supporting the conclusion of a subtle D-dominance in the synthetic allohexaploids.

A heatmap of all libraries showed good data consistency (Fig. 1d). Within the synthetic allohexaploids, reciprocal crosses and different generations originating from the same parents are sometimes intermixed, indicating high similarity of these libraries. The *in silico* karyotype check suggested that two of our transcriptomes were obtained from aneuploid samples (Supplementary Figs. 1–4, Additional file 3). These were the leaf library C1-ABxD-T1 monosomic for the chromosome 5B, and another leaf library C1-DxAB-T2 monosomic for the chromosome 1B. However, the top two principal components (PCs; Fig. 1e,f) did not distinguish these monosomies, suggesting that the transcription is at least partially compensated by the present homolog. On the other hand, the PC2 of the leaf transcriptomes clearly separated four libraries that were sampled in a different year (Supplementary Tables 3, Additional file 2), indicating a technical effect that could interfere with the DE analysis. We therefore decided to exclude these four libraries, but to keep the two monosomic samples, with a note of caution for the comparisons involving leaf transcriptomes of Lx109-C1 and 109xL-C1.

### Changes in gene expression

**Fig. 2:**
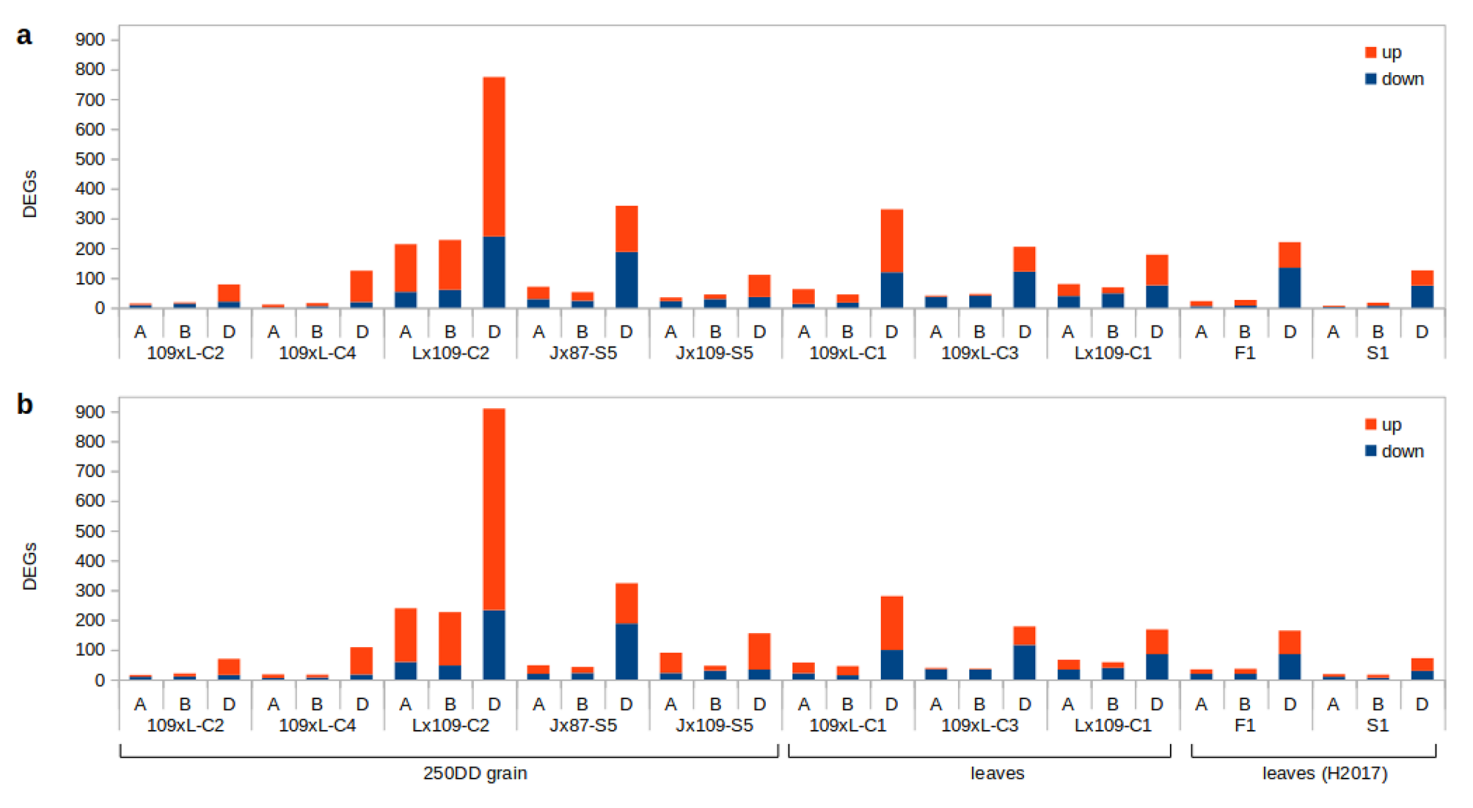
Summary of the DEGs detected in synthetics compared to their parents at the FDR < 0.01 and fold change > 3. **a**: STAR-pipeline, **b**: BOWTIE2-pipeline. H2017 refers to data produced by [29]

**Fig. 3:**
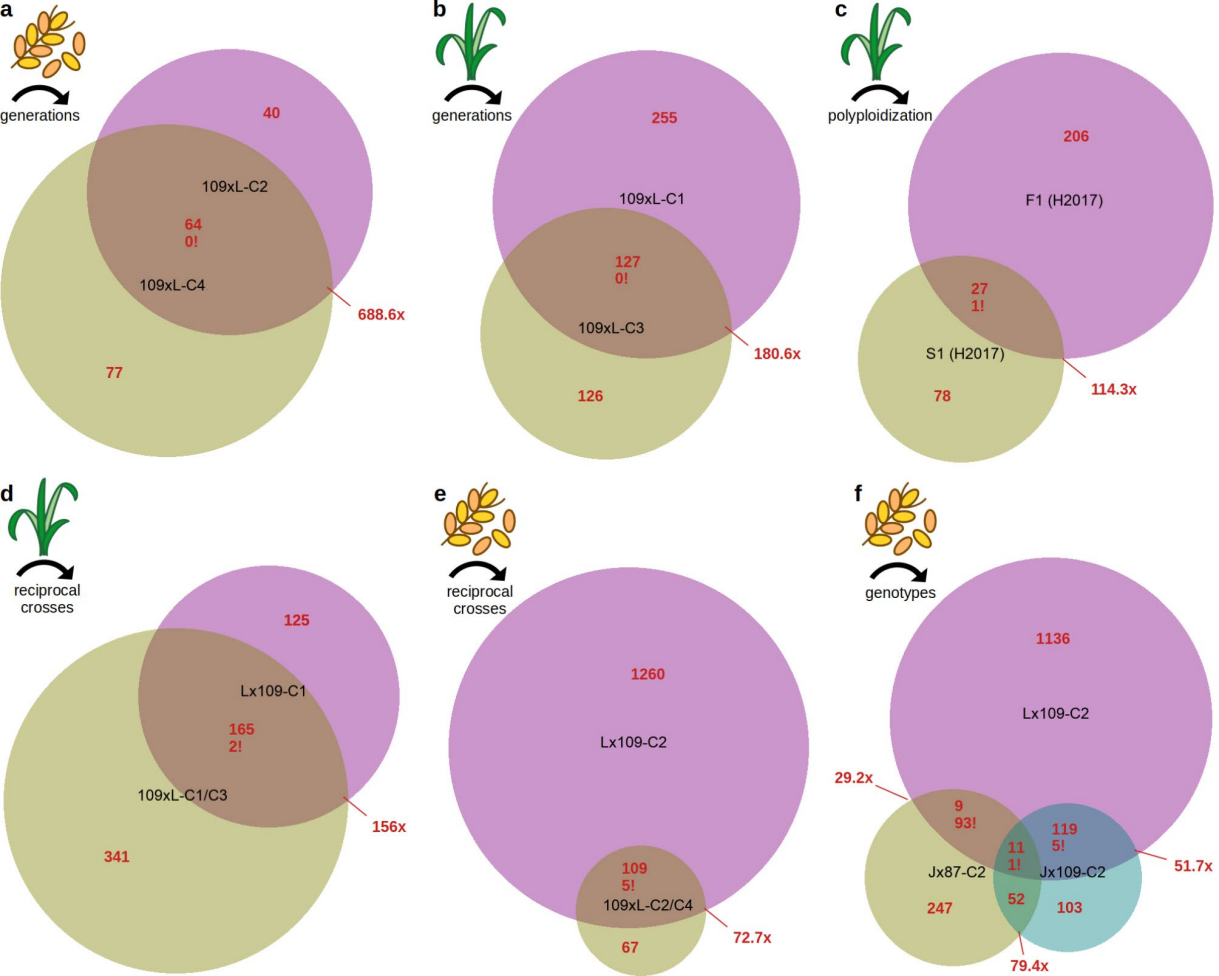
Overlaps between the sets of DEGs identified from comparisons between the parents and the synthetics. The top left corners indicate the biological material and the variable being compared on the Venn diagrams. **a**: A diagram showing two sets of DEGs detected in grain transcriptomes of two synthetic generations, **b**: two sets of DEGs detected in leaf transcriptomes of two synthetic generations, **c**: two sets of DEGs detected in a hybrid and a synthetic leaf transcriptome, **d**: two sets of DEGs detected in leaf transcriptomes of reciprocal synthetics, **e**: two sets of DEGs detected in grain transcriptomes of reciprocal synthetics, **f**: three sets of DEGs detected in grain transcriptomes of different synthetic genotypes. Genotypes’ ID is shown in black in the centre of the circles. DEG counts of different fractions are shown in red, with two numbers for each overlap - the top one showing the number of DEGs with the same direction of gene expression change, and the bottom one followed by an exclamation mark the number of DEGs in contrasting directions. The red numbers outside of the Venn diagrams show over-representation of the intersection in respect to a count expected by chance. All overlaps have high statistical significance, according to exact hypergeometric probability with normal approximation (http://www.nemates.org/MA/progs/overlap_stats.html). In these calculations, the ‘total number of genes’ was the number of genes that are expressed (non-zero read counts) in both of the relevant DEG analyses

Our analysis identified hundreds of robust DEGs (Differentially Expressed Genes) at FDR (false discovery rate) < 0.01 and fold change > 3 (Supplementary Tables 4, Additional file 2; Supplementary Fig. 5, Additional file 3) between the synthetics and their parents, presumably induced by the hybridization or the whole-genome duplication process. Similar DEG numbers per synthetic genotype were observed with both the Bowtie2 (numbers reported below) and STAR pipelines (Fig. 2). There is no statistical difference in the number of DEGs between the synthetics produced with colchicine (C) and through spontaneous chromosome doubling (S). We observed statistically significant overlaps between the sets of DEGs detected in consecutive generations (Fig. 3a-c), and sets of DEGs detected in synthetics from reciprocal crosses (Fig. 3d,e). Assuming these overlaps do not stem from systematic technical biases, the re-occurring DEGs testify to the non-random, and at least partially heritable and reproducible nature of post-polyploidization deregulation of gene expression. Significant overlaps between sets of DEGs were also detected when comparing synthetics originating from different parental combinations (but having the same cross direction) (Fig. 3f). Only eleven genes were found to be de-regulated in the same direction in the three different synthetic genotypes. Moreover, while the DEG overlap across genetically different synthetics (Jx87-S5 vs. Lx109-C2) is statistically strong, most of the shared DEGs differ in the direction of the expression change, being upregulated in one synthetic and downregulated in the other (94 out of 114; Fig. 3f).

We also observed much smaller - but still significant - overlaps between the DEGs detected in the grain and leaves of the same genotype (Supplementary Fig. 6, Additional file 3). Much of this significance dissipated when the direction of the expression change was also considered. This result indicates a lack of consistency in DE across tissues. We checked whether the low number of DEGs shared between the grain and leaves is due to large differences between the grain and leaf transcriptomes in general. This does not seem to be the case, since most genes (including the DEGs) expressed in the leaves are also expressed in the grain (Supplementary Fig. 2). Therefore, the insignificant overlap of DEGs indeed suggests that polyploidization-induced transcriptomic changes are not conserved across different tissues.

Higher numbers of DEGs were consistently detected on the D subgenome across different genotypes, tissues and pipelines. In total, we detected twice as many DEGs on the D subgenome, than we did on the A and B subgenomes together. Total DEG numbers per synthetic genotype vary considerably from 104 (109xL-C2) to 1,374 (Lx109-C2). In the grain tissue, more DEGs were yielded from crosses where durum wheat was used as the maternal parent, analogically to natural bread wheat, as opposed to crosses where the maternal parent was *Ae. tauschii* (on average, 693 and 123 DEGs, respectively). However, the opposite was observed in the leaves (on average, 199 and 318 DEGs, respectively). A dominant direction of gene expression change is also difficult to conclude. While in some synthetic genotypes and tissues, upregulation is far more frequent than downregulation (e.g., 3.05× more upregulated genes in the grain of Lx109-C2), this pattern is reversed in a different genotype (1.24× more downregulation in the grain of J87-S5), or in a different tissue of the same genotype (1.16× more downregulated genes in leaves of Lx109-C1).

In addition to the analysis of trancriptomes produced by us, we also reanalysed RNA-seq data published by Hao et al. [29]. These data had been sampled from leaves of a *T. turgidum* subsp. *turgidum* genotype AS2255, *Ae. tauschii* genotype AS60, their allotriploid hybrid and first polyploid generation. A collection of DEGs we identified in these hybrid and polyploid samples forms a pattern that is consistent with the data produced by us. In particular, most of the DEGs are located on the D subgenome, without clear dominance of down- or up-regulation (Fig. 2), and with significant heritability across generations (Fig. 3c). Our results confirm the previous observation that transcriptomic changes occur already in the inter-specific hybrid, i.e. before the whole-genome duplication [29].

### Gene ontology (GO) analysis of the DEGs

We performed GO analysis for each set of DEGs (i.e. separately for up- and down-regulated DEGs in each parent-synthetic comparison of DE). GO terms significantly enriched in the DEGs from either pipeline (STAR or Bowtie2) were combined, but only GO terms re-occurring across different synthetics are reported here (Supplementary Tables 5, Additional file 2). For genes downregulated in leaves, we detected significant enrichment of several GO terms related to photosynthesis and plastids. The terms photosynthesis, light harvesting, chlorophyll binding, protein-chromophore linkage, response to light stimulus, photosystem I, photosystem II, chloroplast thylakoid membrane, plastid, plastoglobules and thylakoid were all enriched within leaf DEGs downregulated in the synthetics of multiple genetic backgrounds. Some of these GO terms (e.g. photosystem I and chloroplast thylakoid membrane) are also enriched among the DEGs downregulated in grain, indicating that polyploidization can affect the same processes in different tissues. Nonetheless, several over-represented GO terms appear to be tissue-specific. Surprisingly, some GO terms are over-represented in both down- and up-regulated DEGs of the same tissue. For example, enzyme inhibitor activity is enriched among the genes downregulated in the grain of Lx109-C2 and Jx109-S5; but also among genes upregulated in the grain of Jx87-S5, Jx109-S5, Lx109-C2 and 109xL-C2. Among the GO terms over-represented in upregulated DEGs, we found some related to stress responses, in particular, response to water, killing of cells of other organism, defence response to fungus (all detected in grain), and response to jasmonic acid (in leaf).

We have also tested GO enrichment in the 94 DEGs with different direction of gene expression change in the two genetically different synthetics Lx109-C2 and Jx87-S5 (Fig. 3f). This set of DEGs (Supplementary Tables 6, Additional file 2) is significantly enriched in GO terms related to pathogen resistance, specifically defence response to fungus, killing of cells of other organism, chitin catabolic process, polysaccharide catabolic process, protein catabolic process, proteolysis (biological process); extracellular region and cell wall (cellular components); and endochitinase, pectin acetyltransferase, aspartic-type endopeptidase, cysteine-type endopeptidase inhibitor, and acetylserotonin O-methyltransferase activities (molecular function).

### Changes in TE transcription

The analysis described above detected several TE families among the DEGs in the Bowtie2-pipeline. However, a closer inspection revealed that most of the de-regulated TE signals in the DE analysis are found in TE families suffering from the ‘subgenome mismatch’ problem (see Supplementary Note). This is exposed by the RPMs in the parents, where most of the reads are assigned to the wrong subgenome (e.g., TE family RLC_famc6 reads in *Ae. tauschii* were mapped overwhelmingly on the B rather than the D subgenome of the reference), leading to subgenomic counts that are incompatible across ploidy levels. We therefore did not report these TE families on the Fig. 2, nor on the Venn diagrams (Fig. 3).

Moreover, many TE families show significant differences in their overall level of transcription between *Ae. tauschii* and durum wheat (Supplementary Tables 7, Additional file 2). For example, RLG_famc7 transcripts are found in *Ae. tauschii* at RPM levels ~ 500, but they are much more abundant in *T. t.* subsp. *durum* (~ 7,900 RPM). Since we are unable to specifically assign TE-derived reads to the three subgenomes, the appropriate approach is to compare overall levels of transcription between the synthetics and MPV. We have therefore performed an additional DE analysis specifically adapted for the TEs. Unlike in the analysis of gene transcription, here we summed up the A-, B- and D-mapped RPMs (together with RPMs mapped to unassembled contigs) for each TE family. Such sum totals of the synthetics were compared to MPVs calculated for each TE family as 1/3*RPM(*Ae. tauschii*) + 2/3*RPM(*T.t.* subsp. *durum*), using all combinations of parental replicates. Subsequently, the synthetic and midparent RPM datasets were analysed with a GLM in edgeR, using a > 3 fold change and < 0.01 FDR thresholds.

Results show that the transcription level of most TE families in the synthetics is statistically similar to the MPVs (Supplementary Fig. 7, Additional file 3). Nonetheless, several TE families (two families of *Copia* LTR retrotransposons, two families of *CACTA* DNA transposons, and one unclassified LTR retrotransposon family) appear significantly upregulated in some synthetic samples. In particular, RLC_famc4 and RLX_famc22 are upregulated in both Lx109-C2 and Jx109-S5 (grain); RLC_famc24 is upregulated in Jx87-S5 (grain); DTC_famc15 is upregulated in Lx109-C2 (grain); and DTC_famc41 is upregulated in both 109xL-C1 and Lx109-C3 (leaves). These TE families are not among those with significant parental differences (Supplementary Table 7), suggesting that a relaxation of TE silencing is indeed a likely explanation of the upregulation signals in the synthetics.

We have checked whether some TE upregulation signals could be caused by upregulation of overlapping genes and vice versa. We have identified only one upregulated TE family where an element overlaps with an upregulated gene on the same strand. This is a low confidence gene TraesCS5D02G548800LC (upregulated in Lx109-C2 and Jx109-S5) that is entirely located within an RLX_famc22 element. Given that the mapped reads do not follow the CDS structure of TraesCS5D02G548800LC and cover a much larger region of the TE (Supplementary Fig. 8, Additional file 3), it appears that the upregulation signal of the gene is a consequence of the upregulation of the TE family.

Additionally, we examined a possible relationship between DE of genes and their distance to TEs. Both up- and down-regulated genes frequently overlap with a TE, but can also be found kilobases away from the nearest upstream TE, with similar distributions for TEs on the same and opposite strands (Supplementary Figs. 9–16, Additional file 3). In other words, we detected proportional amounts of significantly downregulated genes overlapping with a TE on the same and opposite strands, as well as significantly upregulated genes overlapping with a TE on the same and opposite strands. No particular TE superfamily appears to be associated with either up- or down-regulated genes. In terms of statistics, up- and down-regulation of DEGs is independent of the orientation of the closest TE in all synthetics, both up- and down-stream of DEGs (chi-square test; alpha 0.05). Therefore, the orientation of the closest TE has no relation to whether the gene is up- or down-regulated. Similarly, the distance to TEs does not show a systematic association with the direction of the gene expression change either. The distance to TEs is statistically similar among up- and down-regulated DEGs (T-test, alpha 0.05) in nine out of 16 comparisons (separately evaluating upstream and downstream TEs in eight different synthetics). In the remaining comparisons, upregulated DEGs are significantly closer to TEs in five cases, and downregulated DEGs are closer in two cases.

### Homoeolog expression bias

**Fig. 4:**
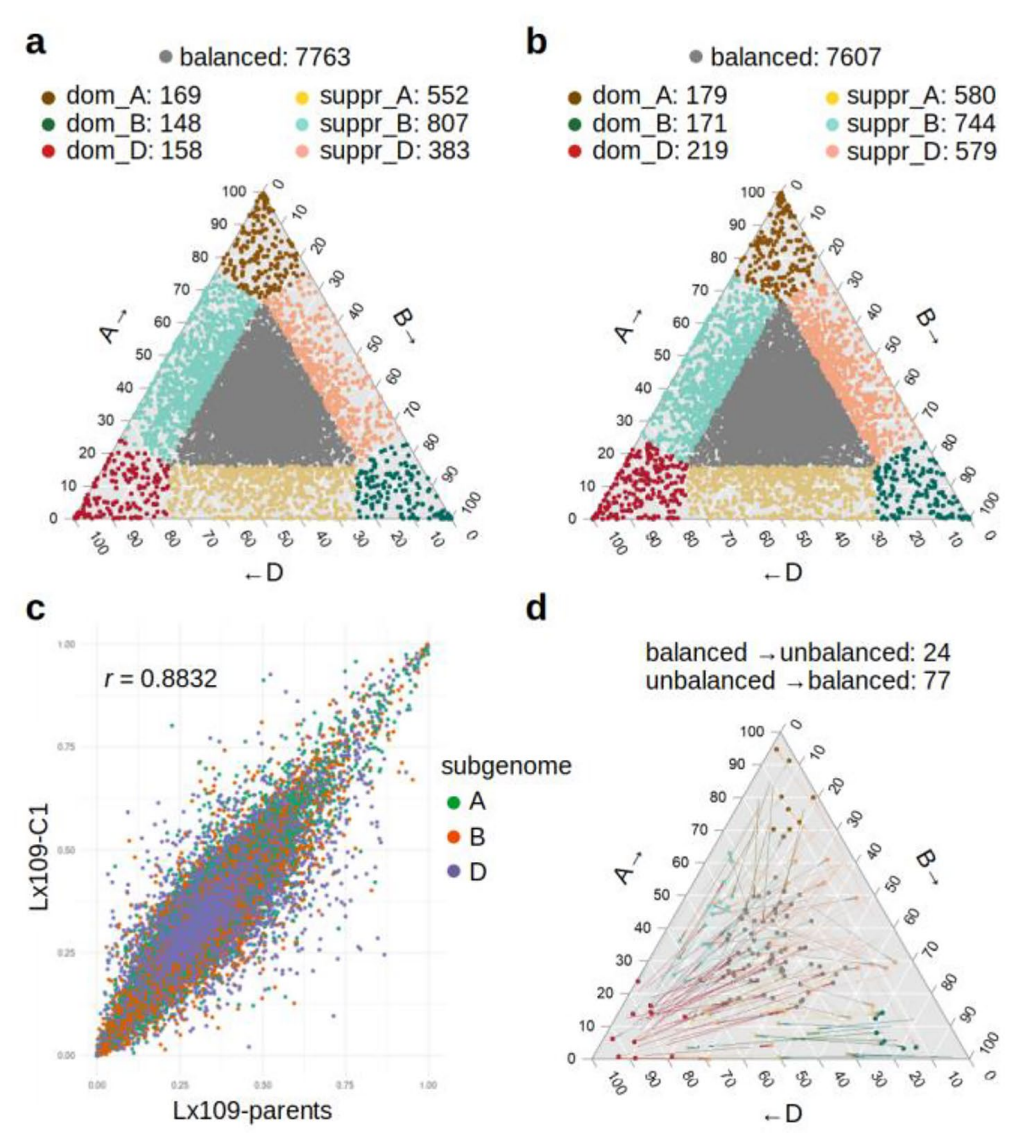
Homoeolog bias and parental legacy. **a**: A ternary plot for Lx109-C1 (leaves). Homoeologs contributing < 0.167 and > 0.666 to the total triad expression are regarded as biased and their triads are divided into several categories. **b**: A ternary plot for the combined parents Langdon and *Ae. tauschii*-109 (leaves), showing similar yet slightly higher levels of homoeolog bias compared to their allohexaploid derivative. **c**: A scatter plot showing the contributions of individual homoeologs to triad expression in the Lx109-C1 synthetic and its parents. **d**: A ternary plot showing only the triads that changed their position (above a 0.3 eigen distance cut-off) as a result of polyploidization. The coloured dots show the triad position in the Lx109-C1 synthetic, and the change in respect to the parents is indicated with the arrows. Grey-coloured arrows indicate that the original triad was balanced, while grey-coloured dots indicate that the new triad position is balanced. Changes that do not involve unbalanced positions, and triads that became activated/inactivated in the synthetic are not shown

In addition to the DE analysis, we conducted an independent analysis of homoeolog bias in the nascent synthetics. Using the ternary plot concept [10], we found that 77.8-78.4% of triads in leaves, and 83.1-84.5% of triads in developing grain have a balanced expression (i.e., are expressed from all three homoeologous loci). These proportions are similar to the natural bread wheat cultivar Recital, with 78.7% and 82.3% of balanced triads in leaves and grain, respectively (Supplementary Fig. 17, Additional file 3). In total, there are 188–207 and 458–475 dominant triads in grain and leaves, respectively (Fig. 4a; Supplementary Fig. 3), without statistical bias towards any subgenome (chi-square test, P > 0.12). However, we observed a statistically strong bias in the number of A-, B- and D-suppressed triads for each synthetic and tissue, as well as for natural bread wheat (p < 10^− 8^ except in the grain of Jx87-S5), with a relative dearth of D-suppressed genes (grain) accompanied with an excess of B-suppressed genes (leaves). This result is consistent with the observation of the subtle D-dominance observed on the basis of total transcription (Fig. 1c).

Next, we addressed the question of homoeolog bias consistency in various comparisons. The relative expression of each gene in a triad can be given as a {0,1} vector (with 0 meaning no contribution to the overall expression of the triad, and 1 meaning that the expression of the triad is entirely supplied by the particular homoeolog). Subsequently, homoeolog bias can be compared across pairs of samples via Pearson’s correlation coefficient and scatter plots (Fig. 4c; Supplementary Fig. 18, Additional file 3). Our results show that homoeolog bias is very stable across generations (correlation coefficient ~ 0.97; Supplementary Fig. 18a,b), and highly consistent patterns are also obtained in reciprocal synthetics (correlation coefficient ~ 0.95; Supplementary Fig. 18c,d). Correlation of homoeolog bias between synthetics and their parents is relatively high (0.8–0.9; Fig. 4c, Supplementary Fig. 18e-l), suggesting that a large part of the observed bias in the synthetics is due to parental legacy. The similarity to the parents is in all cases higher than the similarity to natural bread wheat Recital (0.68–0.74; Supplementary Fig. 18m-q). A much lower correlation was observed across tissues, both in the synthetic and natural allohexaploids (0.54–0.58; Supplementary Fig. 18r-u), indicating that homoeolog bias is largely tissue-specific.

Finally, we investigated the relationship between homoeolog bias, DE and parental legacy. We found that dominant and suppressed homoeologs of synthetics (contribution to the total triad expression > 0.667 and < 0.167, respectively) differ significantly in their polyploidization-triggered expression change (T-test, p < 10^− 5^). The dominant homoeologs are upregulated more frequently compared to the suppressed ones (Supplementary Fig. 19, Additional file 3), indicating that homoeolog bias and DE are not independent. However, the vast majority of unbalanced homoeologs do not show significant change in expression compared to the parents, suggesting parental legacy as the main source of homoeolog bias.

The ternary plots (Supplementary Fig. 17) show the expression bias of triads in respect to an ideal 1:1:1 contribution of homoeologs, not in respect to an observed expression in the parents. The ternary plots therefore should not be understood as showing bias that is triggered by polyploidization. In fact, the level of homoeolog bias of parental transcriptomes combined *in silico* is similar to that observed in the synthetics (Fig. 4a,b). Here, the parental triad position is based on a combination of normalized and scaled parental libraries (0.33*diploid RPMs + 0.67*tetraploid RPMs). Subsequently, we can visualize how the position of triads in the synthetics changed compared to the parents (Fig. 4d, Supplementary Fig. 20, Additional file 3). Using a 0.3 eigen distance cut-off, which is an arbitrary threshold that disregards smaller-scale changes for the benefit of plot clarity, we demonstrate that very few synthetic triads changed their homoeolog bias category as a result of polyploidization. Moreover, in all parents-synthetic comparisons, the triads changed more frequently from unbalanced (in the parents) to balanced (in the synthetics) than they did in the opposite direction (Fig. 4d, Supplementary Fig. 20), and this pattern is insensitive to the distance cut-off. This observation is incompatible with the hypothesis that polyploidization triggers homoeolog expression bias, and supports the alternative - the bias observed in the synthetics is largely due to parental legacy, and the net result of polyploidization is actually a slight increase of balanced triads.

## Discussion

### Extent of the transcriptomic shock and the role of systematic technical biases

A transcriptomic shock, i.e. a rapid and widespread rearrangement of gene expression, is a widely-reported consequence of allopolyploidization. Some microarray studies [24–26], together with the observation of pervasive homoeologous expression bias in bread wheat [10, 11], suggest that transcriptomic shock in wheat is strong. This expectation was corroborated by a transcriptomic study by Hao et al. [29], who found that 1%, 1,2% and 25.2% of A-, B-, and D-located homoeologs, respectively, are differentially expressed in the first allohexaploid generation compared to the parental transcriptomes.

In the present study, we analysed the transcriptomes in eight parents-synthetic comparisons that include different tissues, synthetics produced from different parental genotypes, synthetics produced from reciprocal crosses, and synthetics from consecutive generations. Our DE analysis involved several key features and corrections that are important for an unbiased comparison of transcriptomes across different ploidy levels. These include the use of the full allohexaploid reference for the read mapping of all samples (including the diploids and the tetraploids), a correction of ‘subgenome mismatches’, and separate normalization of the AB- and D-subgenomes (Supplementary note). Additionally, the DE analysis was performed with two alternative read-mapping and read-summarization pipelines.

With a stringent fold change threshold of 3 and FDR < 0.01, we obtained a relatively low number of DEGs in both pipelines. We identified 110-1,224 DEGs per synthetic genotype (STAR-pipeline; Fig. 2a), which translates to only 0.14-1.51% of all expressed high-confidence genes (with ≥ 1 read detected in the compared subset of libraries). Similar numbers of DEGs were identified when low-confidence genes were also considered (104-1,374 DEGs per synthetic; Bowtie2-pipeline; Fig. 2b). Since these results are in a stark contrast with the previous studies, we have included the data produced by Hao et al. [29]. In our pipelines, these data yielded DEGs within the same range as our original samples (Fig. 2a,b). Even after relaxing the DE thresholds (FDR < 0.05, no fold change threshold, as in [29]), we detected only 203 and 261 DEGs (BOWTIE2- and STAR-pipeline, respectively) in the first allohexaploid generation, compared to 4,293 in the original study. This ~ 20x difference in the number of DEGs detected in the same dataset must stem from differences in the analytical procedures. We have identified several critical points that may lead to systematic biases (Supplementary Note) and we concluded that differences in library normalizations have probably the highest impact on these two analyses. The authors of the original study compared the normalized synthetic allohexaploid libraries to the normalized libraries of *Ae. tauschii* and *T. turgidum* (M. Hao, personal communication), without taking into account the interploidy nature of such comparisons. Since the parents and the synthetics have radically different numbers of genes, transcription from a particular gene constitutes very different fractions of the total transcription in the parent and the polyploid, making direct inter-ploidy comparisons incompatible. In contrast, our approach was to split the transcriptomes of all allohexaploids into their AB- and D-parts, and normalize the AB-parts together with the AB parent, and the D-parts with the D parent, effectively avoiding inter-ploidy comparisons. We conclude that the number of DEGs reported previously [29] suffers from inappropriate data normalization, and the actual fraction of genes deregulated in nascent wheat allohexaploids is minor.

Library normalization is not the only potential source of systematic biases in the assessment of the transcriptomic shock. Another problem stems from the high level of similarity between the genomes involved in the polyploidization, combined with differences between the studied genotypes and the reference genome used for the read mapping. This leads to situations where reads are assigned to the wrong subgenome (here referred to as subgenome mismatches), compromising the analyses of homoeolog expression bias and DE [30] (Supplementary Note). Methods developed to explicitly evaluate competing read alignments [31, 32] were found to produce much lower error rates compared to standard approaches, when used in DE analyses across ploidy levels in wheat and *Arabidopsis* [30]. This lead to suspicions that the transcriptomic response to polyploidization has been generally overestimated [12]. Indeed, some recent studies question the very existence of the genomic shock in bread wheat [33], *Arabidopsis* [34] and *Brachypodium* allopolyploids [35], and conclude that the differences from lower-ploidy relatives result from gradual post-polyploidization evolution [34, 35]. Here, we placed special attention to minimize the biases related to read mapping, subgenome mismatches and data normalization, and found that early generations of nascent wheat allopolyploids differ from their parents in a very small fraction of transcripts. This observation questions the validity of the term genomic/transcriptomic shock for the polyploid system investigated here.

### Disproportionate impact on the D subgenome and the dominant direction of expression changes

It has been reported [29] that downregulation of the D-located genes in wheat hybrids/synthetics is by far the most frequent change in the transcription patterns. However, downregulation was not commonly observed as the dominant pattern of non-additive expression in microarray studies, where strong [25] or moderate bias towards upregulation was observed [24, 26, 27]. Here, we report a lack of consistency in the direction of significant gene expression change across tissues, genotypes and cross directions. While upregulation is more frequent than downregulation in the developing grain of most genotypes, the pattern is not retained in leaves (Fig. 2a,b). However, we confirm the observation regarding the disproportionate impact on the D genes. In all our parent-synthetic comparisons, most of the DEGs are located on the D subgenome. The overall ratio of the D-located versus A- and B-located DEGs is 1:0.27 and 1:0.22, respectively (Bowtie2-pipeline), or 1:0.22 and 1:0.23, respectively (STAR-pipeline). The cause of this bias remains unknown, but we can speculate that it relates to the fact that during the allohexaploid synthesis, the D subgenome is added to a species that is already polyploid (*T. turgidum*), with allotetraploidization estimated to have occurred ~ 0.5 million years ago [36]. Expression of genes that are vulnerable to deregulation due to regulatory incompatibilities of the diverged parental genomes has already been altered and stabilised in the AABB tetraploid, while such class of genes in the added D subgenome is still susceptible and therefore impacted by the genome merger.

### Induction of the transcriptomic changes and their heritability

Through the reanalysis of previously published data [29], we confirm that transcriptomic changes occur already in the hybrid (i.e. allotriploid) stage. Only ~ 12% of the DEGs detected in the F1 hybrid were detected as DEGs in the first allohexaploid generation, which also had less DEGs in total (Fig. 3c). This is consistent with earlier findings in wheat [15, 17] and other genera, concluding that the majority of changes observed in allohexaploids had been triggered by hybridization rather than genome doubling [14].

It has also been proposed that genome doubling could actually ameliorate or reverse the transcriptomic changes induced by hybridization, since it had been observed that the number of deregulated genes decreases after polyploidization [29, 37]. We have also observed a marked decrease of DEGs from the hybrid (F1) to the first synthetic stage (S1; Fig. 2), supporting the ‘amelioration by genome doubling’ concept. However, it is not clear whether this is a direct consequence of the doubled genome, or a simple time-dependent adjustment of gene regulation that would have occurred anyway, had the hybrids been fertile. From a comparison of the DEG numbers between the C2 and C4 generations of the 109xL allohexaploids, it appears that the transcriptome does not continue the reversal to the original parental status, i.e. cannot be adjusted further in subsequent generations (Fig. 2), although this cannot be firmly concluded due to the limited number of consecutive generations sampled here.

In summary, transcriptomic changes are triggered by the hybridization step in the polyploid synthesis, with some reversals to the parental expression levels and some additional deregulation in the first polyploid generation. A notable fraction of the transcriptomic changes is heritable across subsequent polyploid generations, both in the grain and leaves. However, the sets of DEGs in leaves and grain do not overlap significantly, despite the fact that most of them are expressed in both tissues (Supplementary Fig. 6). This observation has important implications for the hypotheses of the mechanistic cause of the transcriptomic changes in allohexaploids. In particular, the tissue specificity of DE indicates that the underlying change that manifests itself as a change in transcription is not permanent, but rather plastic. This suggests that the cause is not a rigid alteration of the gene sequence or its methylation profile that would have a uniform consequence across tissues, but rather an alteration of the gene’s regulatory network that is only evident in certain tissues and developmental stages.

### Homoeolog expression bias

We demonstrated that ~ 22% and ~ 16% of triads in nascent wheat synthetics are unbalanced in mature leaves and developing grain, respectively. Similar proportions of unbalanced triads were reported previously for comparable tissues (16.62% for leaf excl. flag leaf; 15.36% for grain milk and soft dough stage of grain development) and data cut-offs (minimum of 5 TPM) [10]. We also confirm that, similarly to DE, homoeolog bias is largely tissue-specific, with hundreds of triads changing their bias category across mature leaves and developing seeds (Supplementary Fig. 20d,e). This is consistent with previous observations across a larger set of sampled tissues [10].

While homoeolog expression bias is well-characterized and rather extensive in natural bread wheat [10, 11], little is known about its origin. One possibility is that the biased expression in syntenic triads results from ~ 10,000 years of evolution since wheat allohexaploidization, as part of the ‘diploidization’ process [38]. However, the observation of homoeolog bias in nascent wheat synthetics (this study and ref. [10]) demonstrates that gradual evolutionary forces are not required for the emergence of biased expression patterns. Early presence of homoeolog bias implies that it is either inherited from the parents, or established immediately after polyploidization as a direct consequence of the triggered gene expression changes (DE).

Whether homoeolog bias is caused by DE cannot be answered by testing the independence of the two phenomena at the gene level, because up-/down-regulation and dominant/suppressed status can be independent even when causally related (e.g., homoeolog dominance can be achieved by upregulation of one homoeolog or downregulation of the other two within the triad). When the independence is tested on the triad level (triads with and without DEGs vs. balanced and unbalanced triads), triads with DEGs are unbalanced more often than expected by chance (chi-square test p < 10^− 10^). However, this test does not reveal the direction of causality, as it is conceivable that parentally unbalanced triads are more likely to be de-regulated in the synthetics, or alternatively, that DE often pushes triads out of their homoeolog balance.

We show that most of the homoeolog bias observed in the synthetics disappears when compared to the parental baseline. Therefore, most of the widespread homoeolog bias observed in wheat synthetics is not caused by the polyploidization, but is merely a continuation of the bias that existed in the parents. A similar conclusion was reached by comparing a resynthesized allopolyploid *Brassica napus* to an *in silico* combination of the parents [39], where parental legacy was found to be the dominant cause of homoeolog bias and asymmetric epigenetic patterns. Additionally, we found that homoeolog bias patterns in natural and synthetic wheat allohexaploids are correlated (Supplementary Fig. 18m-q), and therefore we extrapolate that the homoeolog bias observed in natural bread wheat is also mostly determined by the legacy of its parental genotypes.

### The role of TEs

Regarding the TE activity in general, one must distinguish between changes in TE transcription, and transpositional bursts, since the former does not necessarily lead to the latter. In this study, we focused on the changes in TE expression, and did not attempt to detect new transpositional events. While many TE families have statistically different transcription levels in the diploid and tetraploid parents (Supplementary Table 7), very few TE families in the synthetics differ significantly from the MPV. Out of ~ 500 TE families annotated in the Chinese Spring reference, only five were identified as differentially expressed in one or more synthetics (Supplementary Fig. 3). Notably, all of these were upregulated, and included both LTR-retrotransposons (*Copia*) and DNA transposons (*CACTA*).

While no transposition bursts have been detected in nascent wheat allopolyploids [18, 20, 21], transcriptional activation of the *Wis 2-1 A* retrotransposon has been reported [21], perhaps related to massive alterations of DNA methylation patterns observed for several TE families [19, 20]. Kashkush et al. [21] also reported that silencing of chimeric LTR-gene transcripts was associated with higher levels of antisense transcripts originating from the LTR. It has been proposed that the transcription from the LTR can generate high levels of antisense transcripts related to a neighbouring gene in the opposite orientation, which may be followed by post-transcriptional gene silencing. On the other hand, if a gene is in the same orientation as a TE located upstream, the LTR may provide an alternative promoter, which (if activated in nascent allopolyploids) can cause upregulation of the gene. However, we found that up- and down-regulation of the DEGs detected here is statistically independent from the orientation of the closest TE upstream, which means we cannot support the hypothesis that TE promoters are behind the upregulation of the genes in synthetics. Similarly, up- and down-regulation of DEGs is statistically independent from the orientation of the closest TE downstream, also failing to support the hypothesis that antisense readthroughs are responsible for the silencing of nearby genes. Changes in TEs are therefore not responsible for the observed DE of genes.

These observations in nascent synthetics are consistent with the results obtained from a comprehensive analysis of TEs in the bread wheat genome [33]. Wicker et al. [33] did not find any evidence of a substantial increase of TE insertions following the hexaploidization event, suggesting that TE transcription did not change dramatically after the emergence of natural bread wheat. Similarly to our results on the synthetics, no strong enrichment of a particular TE family was observed in bread wheat upstream of genes in specific expression modules, unexpressed genes or silenced homoeologs, failing to find traces of a genomic shock in gene-TE associations [33].

### Variability of gene expression change across synthetics and implications for wheat breeding

Wheat synthetics have been used extensively, and rather successfully, in wheat breeding programs. Since 1980s, CIMMYT (International Maize and Wheat Improvement Center) has produced over a thousand spring and winter wheat synthetics [40, 41]. These synthetics and their derivatives often carry valuable agronomic traits related to abiotic stress tolerance, biotic stress resistance, grain quality etc., and it has been estimated that a third of all new advanced bread wheat lines for irrigated and low rainfall areas by CIMMYT are synthetic wheat derivatives [41]. However, it has been observed that simple resistance traits of *Ae. tauschii* accessions can be suppressed or diluted in the synthetics derived from them [40], and there can be no correlation between the synthetics and the parental *Ae. tauschii* accessions in complex traits like drought tolerance [42]. This lack of heritability of parental traits in wheat synthetics can be partially caused by epistatic interactions and the (possibly related) polyploidization-induced reprogramming of gene expression. Understanding the mechanism and patterns of transcriptomic changes in allopolyploids is therefore relevant for the exploitation of wheat synthetics in breeding.

Here, we examined two possible sources of gene de-regulation in nascent synthetics, (i) maternal effects and (ii) genotype-specific effects, in order to address the following question. If a gene of interest (e.g., a resistance gene in *Ae. tauschii*) is downregulated in a synthetic produced from a cross where a particular genotype of *T. durum* was used as the maternal parent, is the same downregulation observed in a synthetic from a reciprocal cross, or in a synthetic where a different *T. durum* genotype is used? In respect to (i), we found that the Lx109-C2 synthetic had a much higher number of DEGs in the developing grain compared to the reciprocal 109xL-C2 synthetic (Fig. 2). However, this pattern was not observed in the leaf transcriptome, where Lx109-C1 had fewer DEGs compared to 109xL-C1 (Fig. 2); hence, we cannot conclude that the extent of transcriptional reprogramming is related to the direction of the cross. Moreover, the consistency of gene deregulation among reciprocal cross directions is similar to the consistency across generations, i.e. the cross direction is unlikely to reproducibly change the set of deregulated genes.

An unexpected pattern was observed for DEGs shared across synthetics originating from different genotypes (ii). While Lx109-C2 and Jx87-S5 share 114 DEGs (p < 2.1e-130; exact hypergeometric probability), 94 of these genes changed their expression in the opposite direction (Fig. 3f), which is extremely unlikely to be obtained by chance (p < 8e-15; binomial test). Similar outcomes were found with the STAR pipeline and without the correction of subgenome mismatches (88 and 84 contrasting DEGs, respectively), leading us to conclude this is a genuine result. In other words, we found a set of genes - almost all of them from the D subgenome - that got upregulated in one synthetic as a result of polyploidization, but downregulated in a synthetic of different parental genotypes. This set of DEGs (Supplementary Table 7) is significantly enriched in GO terms related to pathogen resistance. We can speculate that these D-located genes are co-regulated, perhaps via one or a few transcription factors that interact differently in alternative combinations of the AB- and D-parents, leading to different direction of expression change in the synthetics. Such genotype-specific, trans (inter-subgenome) interactions between regulatory elements and transcription factors can explain the lack of correlation between the traits expressed in synthetics and their parents [42], but our observation also suggests that alternative AB-parents could be useful in breeding when a desired trait of *Ae. tauschii* is not transferred to its polyploid progeny.

## Conclusions

Based on the RNA-seq analysis of three different synthetic genotypes and their parents, we conclude that only ~ 1% of genes significantly change their transcription levels as a result of polyploidization. The D-subgenome is more affected, compared to the A- and B-subgenomes. The triggered changes are reproducible, partially heritable and identical genes are often affected in different genotypes. However, the up- and down-regulation patterns are not conserved across tissues. The polyploidization-driven gene expression changes can disturb the balance of the affected triads, nonetheless, homoeolog expression bias is mostly determined by parental legacy, and can be actually reduced in polyploids. While several TE families can increase their overall transcription levels after polyploidization, these changes are most probably inconsequential for TE proliferation or the expression of neighbouring genes. Overall, we conclude that synthetic wheat allohexaploids with a genomic composition analogical to bread wheat experience little transcriptional reprogramming as an immediate consequence of polyploidization. This conclusion improves our understanding of the evolution of genome organization in bread wheat and other allopolyploids, and has practical implications for the utilization of synthetic wheat in breeding programs.

## Methods

### Plant material

Seeds of all genotypes used in this study were obtained from the Centre de Ressources Biologiques Céréales à paille (Small grain cereals Biological Resources Centre), INRAE, Clermont Ferrand, France. These included the *T. aestivum* L. cultivar ‘Recital’ (AABBDD), synthetics JOY87 and JOY109 (both AABBDD), and all parental genotypes involved - tetraploid *T. turgidum* subsp. *durum* cultivars ‘Langdon’ and ‘Joyau’ (AABB) and two diploid genotypes of *Aegilops tauschii* ‘Tauschii-109’ (subsp. *strangulata*; the subspecies being the presumed donor of the D-genome in bread wheat) and ‘Tauschii-87’ (subsp. *tauschii*). The synthetics JOY87 and JOY109 had been produced at INRAE - Agrocampus Rennes by spontaneous chromosome doubling in the hybrids from crosses ♀Joyau × ♂*Ae.tauschii-*109; ♀Joyau × ♂*Ae.tauschii-*87 [18]. Two additional synthetics were prepared by us: ♀Langdon × ♂*Ae.tauschii-*109 and ♀*Ae.tauschii-*109 × ♂Langdon. This involved manual castration of the female parents, rescue of the hybrid embryo followed by *in vitro* regeneration, and induction of chromosome doubling by colchicine treatment - all according to standard protocols used at our research unit. The selection of crosses was such that for the cross direction analogical to bread wheat (AABB as the maternal parent), we analysed synthetics originating from the same AABB parent but different DD parents, and synthetics originating from different AABB parents but the same DD parent. Additionally, for the Langdon × *Ae.tauschii-*109 combination, we compared synthetics from reciprocal crosses and two different generations. All plants were subjected to vernalization for eight weeks at 4ºC (8 h day / 16 h night illumination regime).

We named the samples according to the parental combination (e.g., Lx109, indicating by convention that Langdon was used as the maternal and *Ae. tauschii*-109 as the paternal parent), with the polyploid generation appended with ‘C’ (for colchicine-induced polyploids) or ‘S’ (for spontaneous chromosome doubling). In particular, seeds harvested after the colchicine-induced polyploidization represent the first polyploid generation (labelled C1). Plants developed from those seeds were considered to be the same generation (C1), while seeds of those plants were regarded as C2. Therefore, when leaf tissue and developing grain from the same plant are analysed, they correspond to C1 and C2 generations, respectively. In this sense, we sampled vigorous leaves at the anthesis/grain milk stage of the C1 and C3 generation, and developing grain at the 250 degree-days (250DD) post-anthesis, corresponding to C2, C4 and S5 generations. All sampled plants were grown in a growth chamber, alternating 16 h of light at 21ºC and 8 h of darkness at 15ºC. A minimum of two biological replicates (tissues from different plants representing the same genotype and generation), sometimes accompanied with technical replicates (the same biological sample subjected to two RNA extractions) were collected. Collected samples were frozen in liquid nitrogen and stored at -80º. Detailed characterization of all samples is provided in Supplementary Table 3.

### RNA extraction and sequencing

Multiple grains/leaves per biological sample were crushed into powder using liquid nitrogen, mortar and pestle. Up to 1 g of the powder was dissolved in 4.5ml of an extraction buffer (0.1 M NaCl, 10mM Tris-HCl, 1mM EDTA, 1% w/v SDS). Nucleic acids were extracted twice with 3ml phenol:chlorophorm:isoamylalcohol (25:24:1), and precipitated by adding 3 M sodium acetate (1/10 of the sample volume) and two volumes of absolute ethanol. After centrifugation and resuspension of the nucleic acids in RNase-free water, the samples were purified as follows. Between 50 and 75 µg of nucleic acids were treated with 6.8 units of DNase I using manufacturer’s buffers and columns (RNase-free DNase set; Qiagen) and 1 g of glycogen. Eluted RNA samples were stored at -80º and shipped on dry ice to Integragen (Evry, France), where polyA libraries were prepared with the TruSeq Stranded mRNA Sample Prep (or NEBNext Ultra II mRNA-Seq) protocol and sequenced on a HiSeq4000 (or NovaSeq6000) sequencer (Illumina) to obtain ~ 60 million PE150 pairs per sample.

## Read processing and transcript quantification

Raw RNA-seq reads produced in this study (62 libraries) or previously ([29]; Sequence Read Archive IDs SRR3474187, SRR3406932, SRR3474179, SRR3474185, SRR2474176, SRR3474199, SRR3474194, SRR3474201) were processed with Trimmomatic [43], removing adapters, trimming low-quality regions and retaining only paired reads with a minimum length of 36 nucleotides each. Subsequently, two different read mapping and summarization pipelines were used for all samples. The ‘STAR-pipeline’ employed the intron-aware STAR aligner [44] to map the trimmed reads onto a merged reference consisting of the assemblies of the *durum* cultivar Svevo v2 [45] and *Ae. tauschii* subsp. *strangulata* AL8/78 v4.0 [46], removing reads with non-canonical intron motifs and reads with > 3% mismatches against the reference. The complete ABD reference was used for all samples (including the tetraploid and diploid parents; see Supplementary Note). Reads mapping to high-confidence genes were summarized with featureCounts [47], counting individual reads except multi-mapping reads (reads having multiple equally-good mapping choices in the reference genome) and multi-overlapping reads (reads overlapping multiple annotated features). We considered this pipeline to be optimal for the analysis of high-confidence genes, since the mapping procedure accounts for introns and the reference genome used is genetically closest to our plant material. However, this pipeline cannot evaluate the transcription of TEs, since TEs are not annotated on the Svevo assembly. To compensate for this shortcoming, we also mapped all datasets onto the Chinese Spring assembly. Since TE-originating reads frequently map to multiple genomic locations with high presence-absence variation within species, quantification of the transcription of individual TE copies is usually not possible. Instead, TE transcription can be summarized on the family level, revealing the overall expression of annotated TE families. This approach requires specific handling of the multi-mapping reads, which should be neither disregarded nor over-counted. An optimal solution is to assign the multimappers to a single location randomly chosen from the best hits. Such random reporting of multimappers in combination with the paired-read mode gives the best quantification of TE families, according to the best practices for TE analysis from high-throughput data [48]. These settings are possible in both STAR (recommended by [48]) and Bowtie2 [49], which is the most popular tool in the analysis of TE transcription [50–55]. Since Bowtie2 has mapping percentages and true positive rates similar to STAR [48], but an order of magnitude lower memory requirements (an important consideration in the case of the wheat genome), we chose Bowtie2 for our analysis of TE transcription. We mapped the trimmed reads onto the Chinese Spring assembly v1.0 [56] using the --very-sensitive-local mode and only mapping properly paired reads (--no-mixed --no-discordant). Fragments (i.e. concordant read pairs) mapping to high- and low-confidence genes (annotation v1.1) and TEs (annotation v1.0) were summarized on a meta-feature level with featureCounts, counting multi-overlapping fragments fractionally. Prior to read counting, the TE annotation file was edited to represent each individual TE as a feature and each TE family as a meta-feature (i.e., each TE’s ID was reduced to the family level, e.g. RLC_famn30), resulting in per-family read summarization. Features and parameters of both pipelines are compared in Supplementary Table 2.

### ***In silico*** karyotype check and data consistency checks

Euploidy of karyotypes was determined *in silico* for each polyploid sample on the basis of median transcription over large chromosomal fragments. For this purpose, raw read counts produced by the STAR-pipeline were converted to TPMs (transcript per million). Lowly expressed genes with TPM > 0.01 in less than two samples were removed, analysing seed and leaf transcriptomes separately. Parental genotypes (Langdon, Joyau, *Ae. tauschii*-87, *Ae. tauschii*-109) were presumed to be euploid, and their TPMs were averaged across replicates and used as a reference. Median TPM values were calculated for windows of 200 position-ordered genes for each polyploid sample and the reference. Corresponding median values were then compared in a ratio (sample/reference), and the resulting ratios along each chromosome were plotted on graphs. Under the expectation of euploidy, the obtained ratios should be close to one. Aneuploidy or smaller scale changes (e.g., loss of larger chromosomal fragments) should be indicated by values deviating from one (~ 0.5 for monosomy; ~1.5 for trisomy). Additionally, data consistency was checked via a clustering heatmap and a principal component analysis (PCA). The PCA was computed with RPMs in edgeR. For the heatmap, a matrix of Pearson’s correlation was calculated from log-transformed RPMs (bowtie2-pipeline; increased by + 0.001 to enable the transformation) for all pairwise combinations of the samples, and the data was clustered with the UPGMA algorithm.

### Differential expression analysis

Prior to the DE analysis, a correction of ‘subgenome mismatches’ (*Ae. tauschii* reads mapped to the AB chromosomes of the reference, and *T. turgidum* subsp. *durum* reads mapped to the D-chromosomes of the reference) was applied (see Supplementary Note). Briefly, read counts of the parents were converted to RPMs and scaled by the factors 1/3 and 2/3 for *Ae. tauschii* and *T. turgidum* subsp. *durum*, respectively. Subgenome mismatches of one parent were then averaged across biological replicates and the averages added to the corresponding read counts of each library of the other parent.

Additionally, TE-assigned read counts were corrected in the parental samples in the Bowtie2-pipeline. Due to the multimapping nature of most TE-derived reads and the preferred random reporting of a single mapping location (which can be on any subgenome, in both parents and the synthetics), approximately equal counts of TE reads were observed on the A, B, and D subgenomes, even for *Ae. tauschii* and *T. turgidum* subsp. *durum*. However, all TE reads in *Ae. tauschii* and *T. turgidum* subsp. *durum* originate necessarily from the D and AB subgenomes, respectively. And since the summarization of the TE reads is performed on the family level (not on the level of individual TE copies), the read counts assigned to the ‘wrong’ subgenome (the AB and D subgenomes in the case of *Ae. tauschii* and *T. turgidum* subsp. *dicoccum*, respectively) can be added to the per-family read counts of the appropriate subgenome. Accordingly, the AB-assigned TE read counts in *Ae. tauschii* were added to the D-assigned TE read counts, while 1/2 of the D-assigned TE read counts in *T. turgidum* subsp. *durum* was added to each of the A- and B-assigned TE read counts. Similarly, the TE reads mapped to the unassigned contigs of the Chinese Spring reference genome (chrUn) were added to the TE read counts of the appropriate subgenomes in both the synthetic and the parental data sets.

After the corrections, TMM normalization (Trimmed Mean of M-values) [57] was performed using the calcNormFactors function in the edgeR package. Only identical genotypes were normalized together. For example, the D-subgenomes from the synthetic samples Lx109-C2, 109xL-C2 and 109xL-C4 were normalized together with the D parent *Ae. tauschii*-109; and a separate normalization was performed for the AB-subgenome of these synthetic samples together with the AB-parent Langdon. After the normalization, genes with zero read counts across all samples were removed. Identification of DEGs between the parents and the synthetics was performed in the edgeR package, using the GLM implementation [58]. The p-values of differential expression were adjusted by the Benjamini-Hochberg method, and genes with FDR < 0.01 and fold change difference > 3, i.e. log(fold change) > 1.585 and <-1.585, were considered to be differentially expressed.

Analysis of gene ontology (GO) was performed on the Triticeae-Gene Tribe website [59]. In each GO analysis, a customized gene background was used, consisting of expressed genes (non-zero total read count) in the relevant combination of samples. A Benjamini-Hochberg correction was used on the obtained p-values, and terms with FDR < 0.01 were considered significantly enriched.

### Homoeolog expression bias

Our Bowtie2-pipeline was further adapted for the analysis of homoeolog bias. The bam files produced by Bowtie2 were further filtered with Samtools to remove all alignments with mapping quality < 10 (removal of multimappers). Subsequently, fragments mapping to high- and low-confidence genes (annotation v1.0) were summarized as above. The list of homoeologous genes (Chinese Spring v1.0) was retrieved from [10] and used to construct a set of triads. For each gene, we calculated mean expression (RPM) across replicates and removed triads where the total expression of A-, B- and D-homoeologs was below 5 RPM. For each triad, we calculated the contribution of A-, B- and D-homoeologs (on a 0–1 scale) to the total expression of the triad, and we used these fractions to construct ternary plots. We calculated eigen distances from each triad to the vertices, the edge midpoints and the centre of the ternary plot. For example, triad distance to a total subgenome A dominance vertex (i.e. 100% of triad expression comes from the A-homoeolog) was calculated as

1 $$\sqrt{{\left(1-A\right)}^{2}+{\left(0-B\right)}^{2}+{\left(0-D\right)}^{2}}$$

where *A*, *B* and *D* represent the contributions of the A-, B- and D-homoeologs, respectively. Based on the smallest eigen distance, each triad was classified as ‘Balanced’, ‘Dominant A’, ‘Dominant B’, ‘Dominant D’, ‘Suppressed A’, ‘Suppressed B’ or ‘Suppressed D’. For across-sample comparisons, Pearson’s correlation coefficient was calculated for the A-, B- and D-contributions to all triads, and the contributions of corresponding genes from two samples were visualized on scatter plots. Additionally, we represented the change of a triad position on a ternary plot by calculating differences between eigen distances:

2 $$\sqrt{{\left({A}_{1}-{A}_{2}\right)}^{2}+{\left({B}_{1}-{B}_{2}\right)}^{2}+{\left({D}_{1}-{D}_{2}\right)}^{2}}$$

where *A*_*1*_, *B*_*1*_, *D*_*1*_, *A*_*2*_, *B*_*2*_ and *D*_*2*_ represent the contributions of A-, B- and D-homoeologs to the total triad expression in samples 1 and 2, respectively. For clearer representation, we only show triads that changed their homoeolog bias category and moved by > 0.3 eigen distance (arbitrary threshold to highlight only substantial changes). Triads considered expressed in one synthetic and not expressed in another one were not visualised.

## Electronic supplementary material

Below is the link to the electronic supplementary material.

Supplementary Material 1

Supplementary Material 2

Supplementary Material 3

## Data Availability

The datasets supporting the conclusions of this article are included within the article and its additional files. Raw RNA-sequencing data sets are available in the Sequence Read Archive repository, [BioProject ID: PRJNA908799, https://www.ncbi.nlm.nih.gov/sra/].
