# Supplementary material for: Low impact of polyploidization on the transcriptome of synthetic allohexaploid wheat
Source: BMC Genomics. 2023 May 11;24:255. doi: 10.1186/s12864-023-09324-2 (PMC10173476; doi:10.1186/s12864-023-09324-2)
Supplement: Supplementary file 3 — Supplementary Material 3 [file 12864_2023_9324_MOESM3_ESM.docx]

**Low impact of polyploidization on the transcriptome of synthetic allohexaploid wheat**

Meriem Banouh, David Armisen, Annaig Bouguennec, Cecile Huneau, Mamadou Dia Sow, Caroline Pont, Jerome Salse, Peter Civan

**Additional file 3: SUPPLEMENTARY FIGURES**. A document (.docx) containing supplementary figures 1-20. with figure captions.


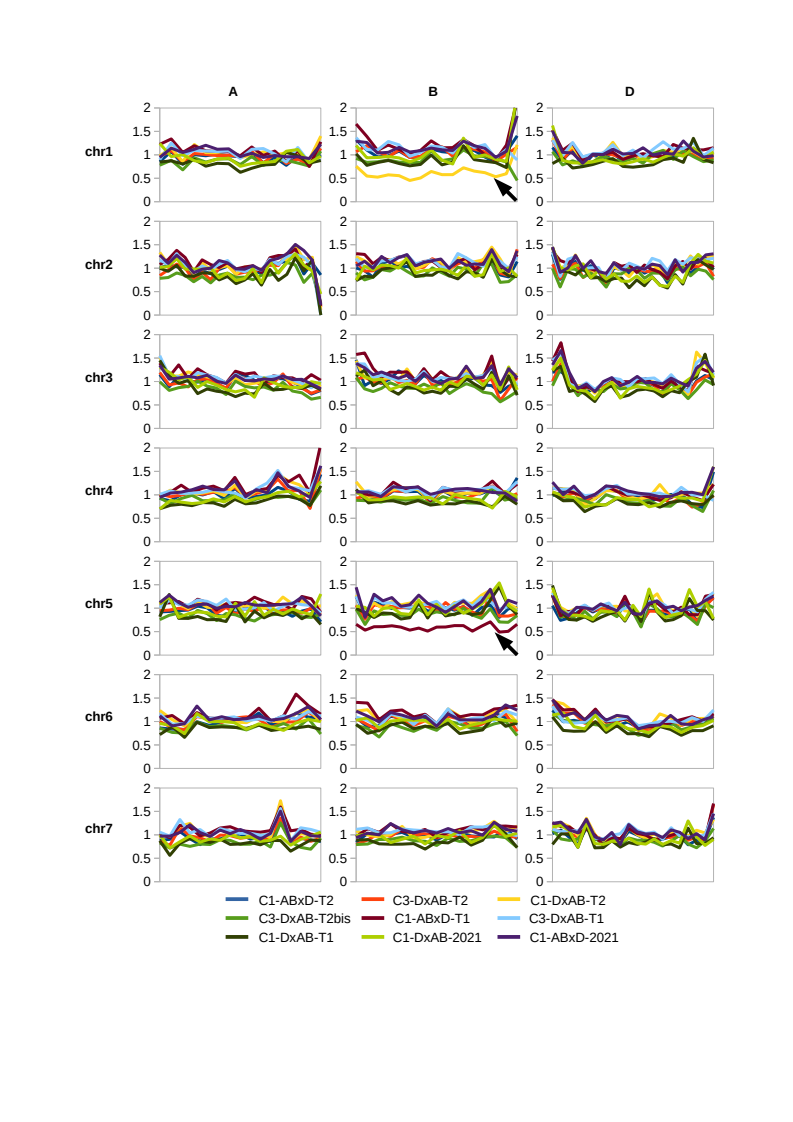
**Supplementary fig. 1** *In silico* karyotypes obtained from the leaf transcriptomes. Monosomic signals are indicated with arrows. The library names correspond to sample designations in the main file, with the suffix indicating biological replicates (e.g. -T1, -T2). To link library IDs with samples, see Supplementary table 1.


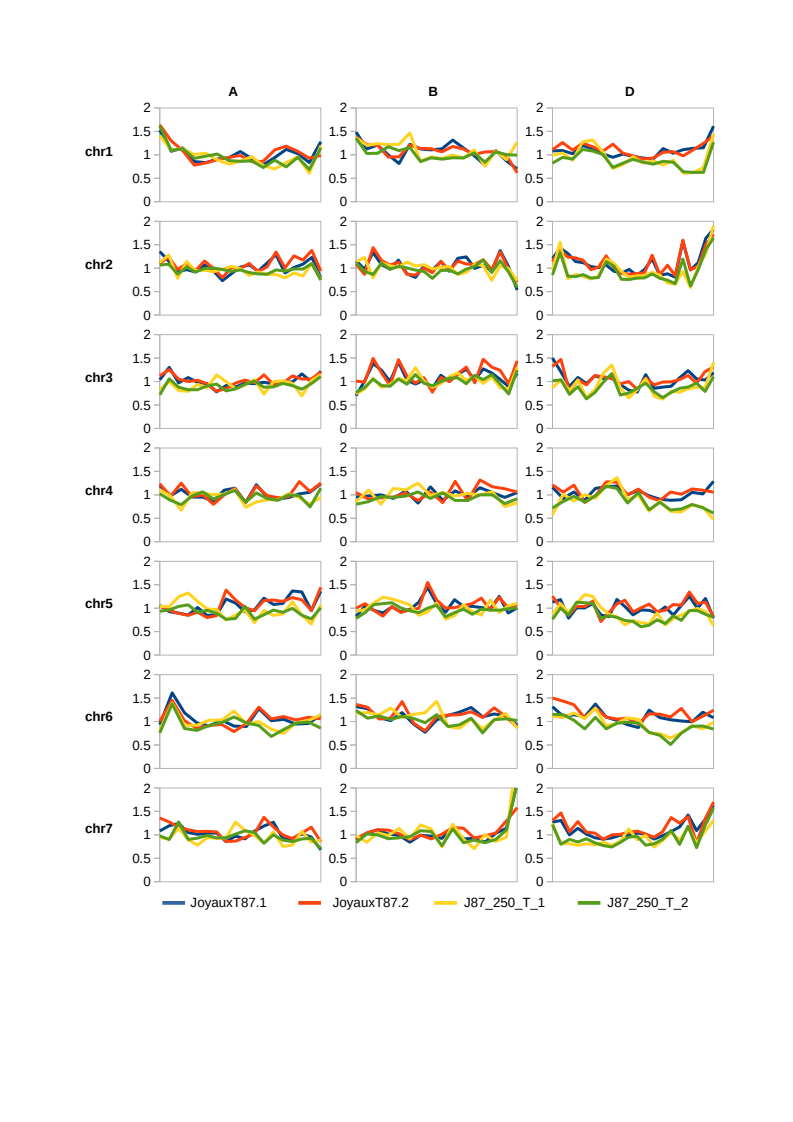


**Supplementary fig. 2** *In silico* karyotypes obtained from the grain transcriptomes of the Joyau×*Ae.tauschii*-87 synthetics. To link library IDs with samples, see Supplementary table 1.


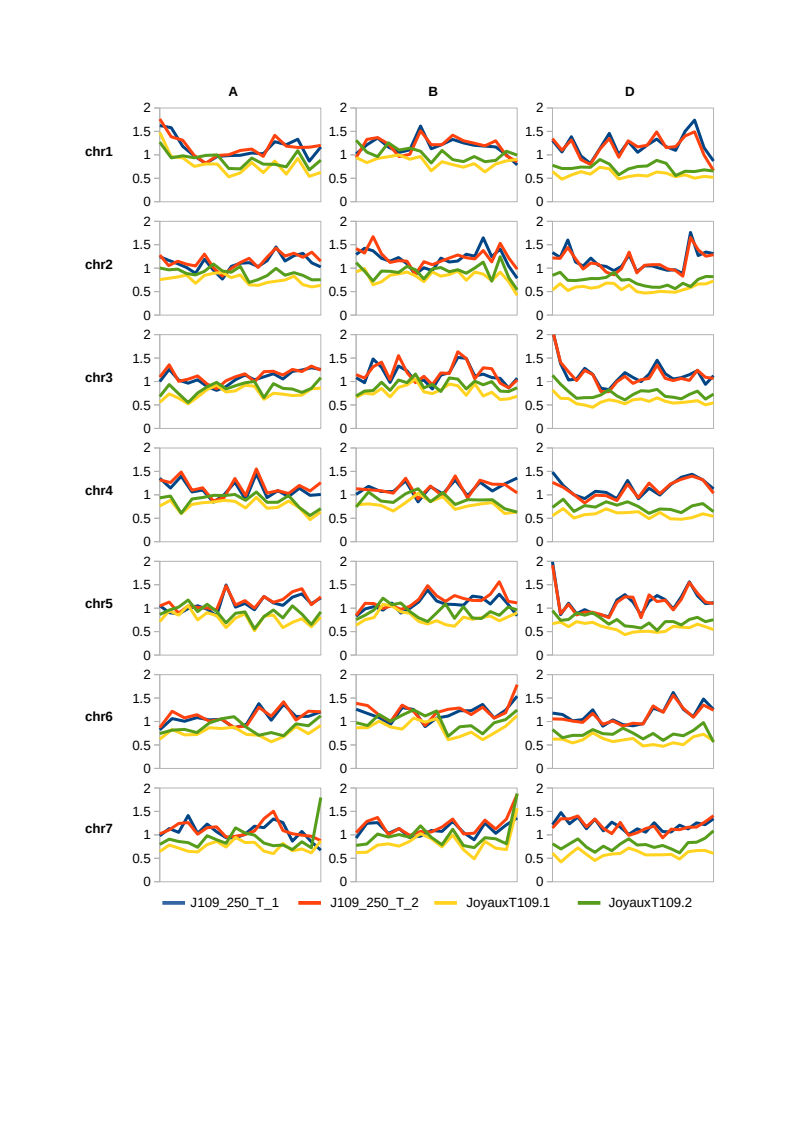


**Supplementary fig. 3** *In silico* karyotypes obtained from the grain transcriptomes of the Joyau×*Ae.tauschii*-87 synthetics. To link library IDs with samples, see Supplementary table 1.


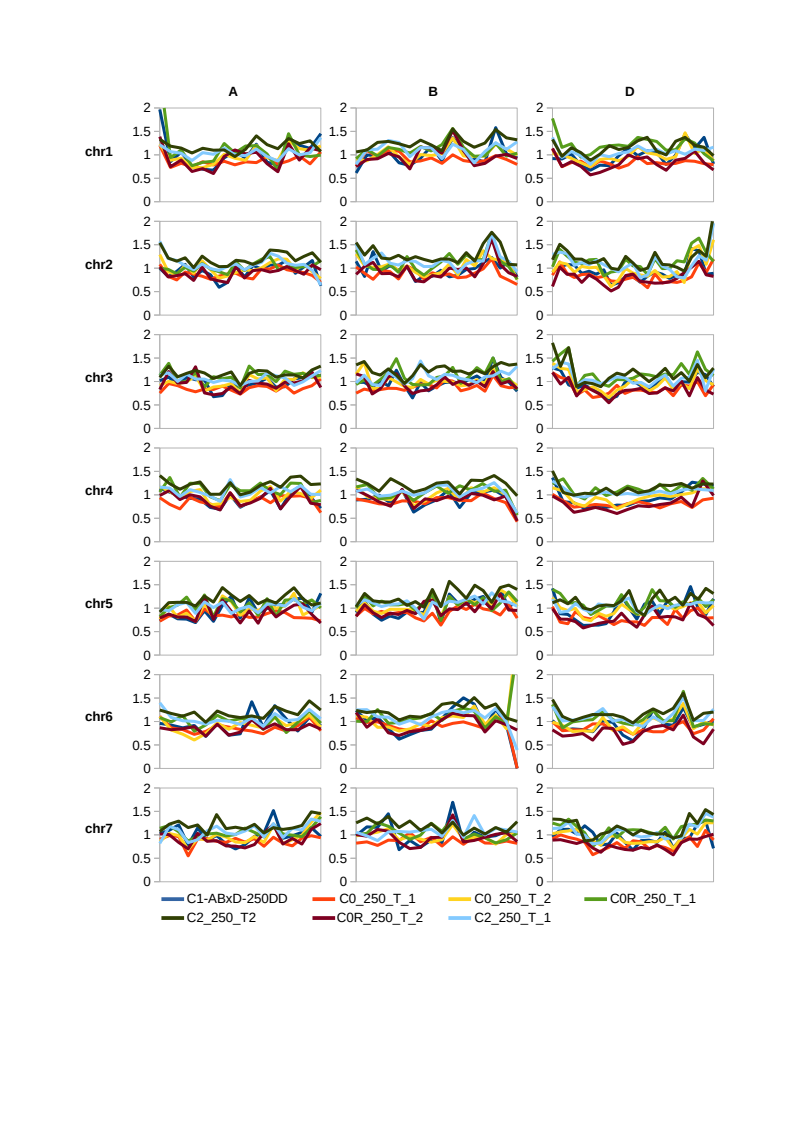


**Supplementary fig. 4** *In silico* karyotypes obtained from the grain transcriptomes of the Langdon×*Ae.tauschii*-109 and *Ae.tauschii*-109×Langdon synthetics. To link library IDs with samples, see Supplementary table 1.


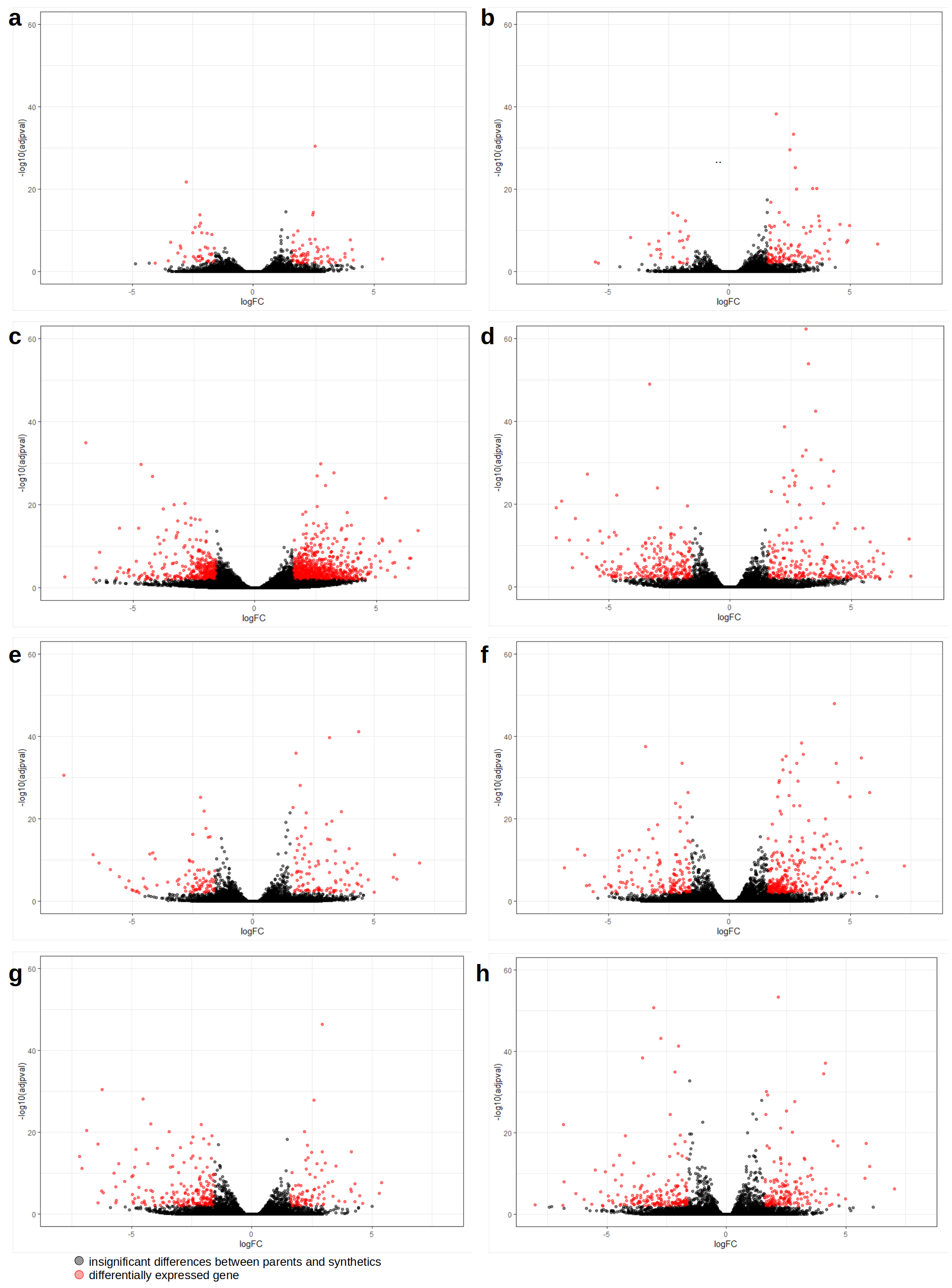


**Supplementary fig. 5** DE analysis between parents and synthetics. **a**: 109xL-C2 vs. parents, **b**: 109xL-C4 vs. parents, **c**: Lx109-C2 vs. parents, **d**: Jx87-S5 vs. parents, two DEGs not shown (exceed the y-axis range), **e**: Jx109-S5 vs. parents, **f**: 109xL-C1 vs. parents, **g**: 109xL-C3 vs. parents, **h**: Lx109-C1 vs. parents, one DEG not shown (exceeds the y-axis range).


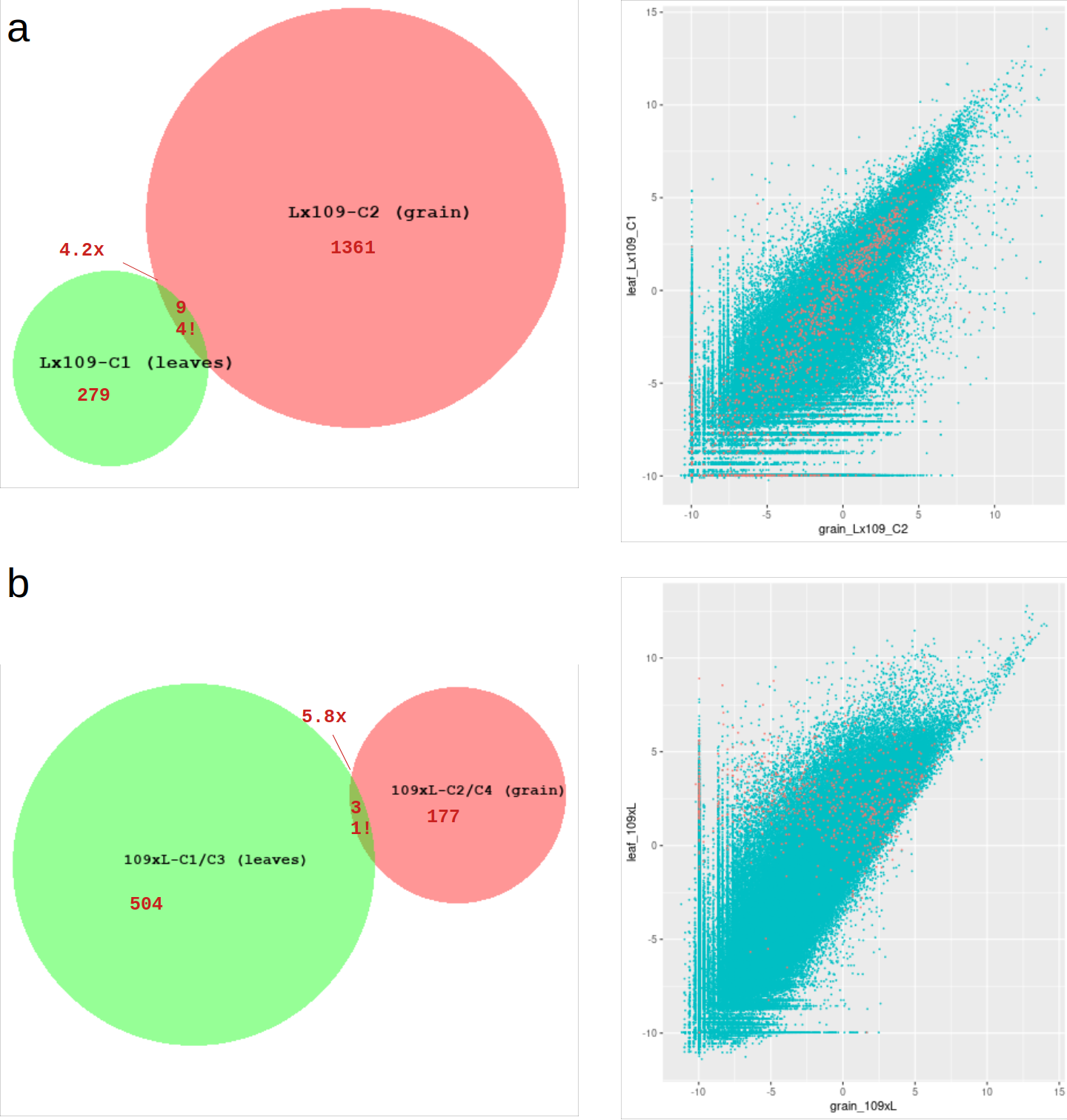


**Supplementary fig. 6** Across-tissue overlaps between the sets of DEGs identified from comparisons between the parents and the synthetics. The left panels show Venn diagrams of the DEGs (see Fig. 3 for more details); the right panels show a joint distribution of the expression of all genes in the grain (x-axis) and leaf (y-axis) of the given synthetic genotype. The data points are log2-transformed RPM values averaged across replicates (zeroes replaced with 0.001 before the transformation). The union of the DEGs shown on the left panels are highlighted in red on the right panels. **a**: Lx109-C2 (grain) vs. Lx109-C1 (leaves), **b**: a union of 109xL-C2/C4 (grain) vs. a union of 109xL-C1/C3 (leaves).


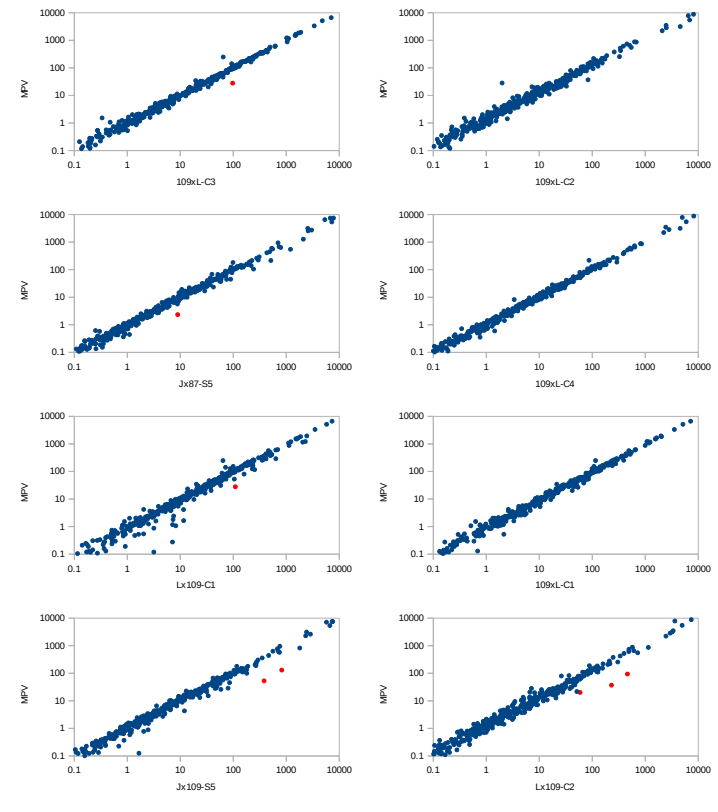


**Supplementary fig. 7.** Transcription of TE classes in the synthetic wheat compared to midparent values. Transcription levels are expressed as RPMs. Each dot on the scatter plots represents a TE class; significant differences between the synthetics and MPVs are highlighted in red.


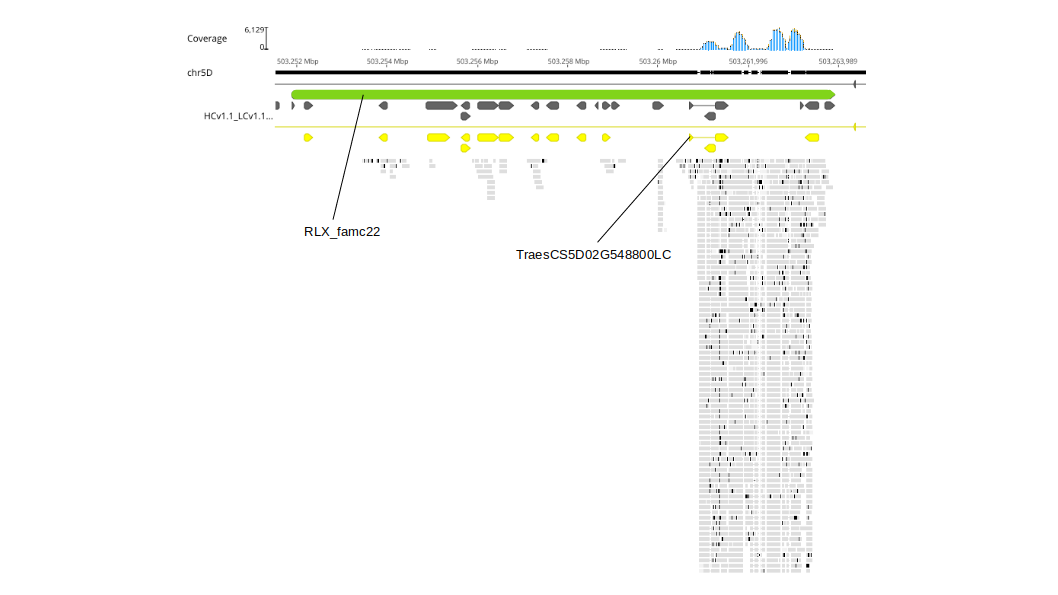


**Supplementary fig. 8** RNA-seq reads mapped to a TE element from the upregulated class RLX_famc22 overlapping with the upregulated gene TraesCS5D02G548800LC. Exons are annotated as dark-grey arrows, CDS in yellow and TEs in green; mapped RNA-seq reads are shown as light-grey bars. The figure was produced in Geneious R11 (https://www.geneious.com).


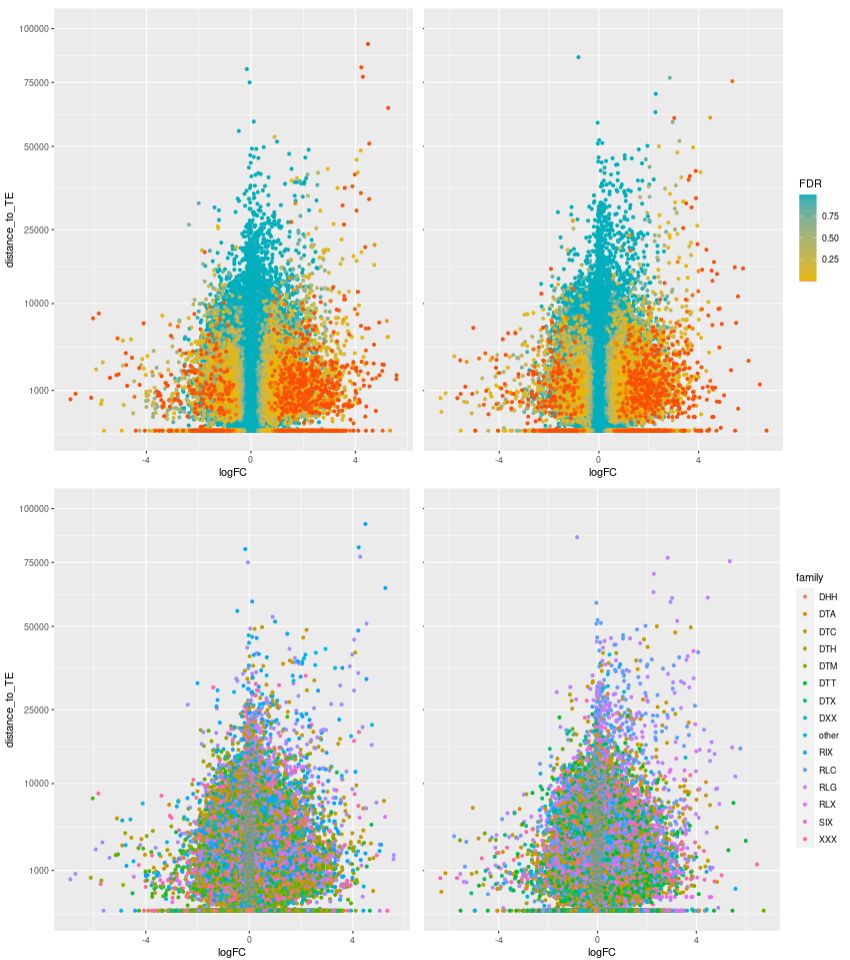
**Supplementary fig. 9** The relationship between up- and down-regulation of genes and their distance to closest TEs in Lx109-C2 (grain). The scatter plots show distance of genes to the closest upstream TE located on the same (left panels) or opposite (right panels) strands. The x-axis shows the fold change results (in respect to the parents) of the DE analysis for each gene, with the colour indicating FDR (top panels), or the superfamily of the closest upstream TE (bottom panels).


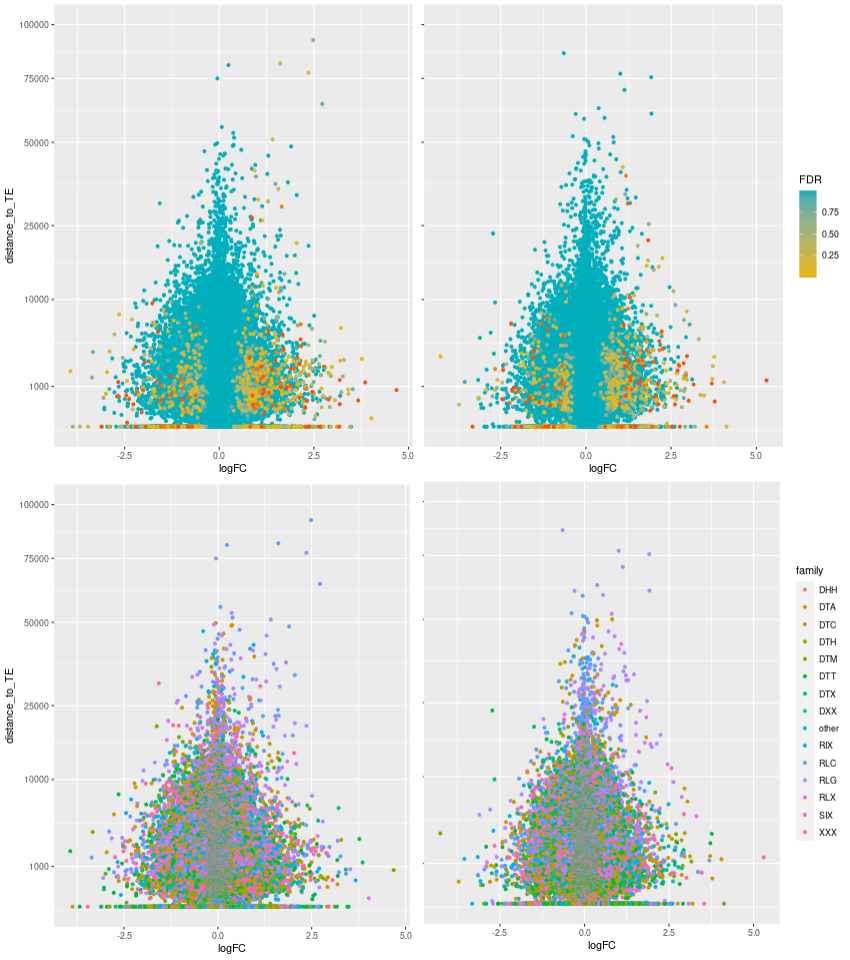
**Supplementary fig. 10** The relationship between up- and down-regulation of genes and their distance to closest TEs in 109xL-C2 (grain). See Supplementary fig. 9 for more details.


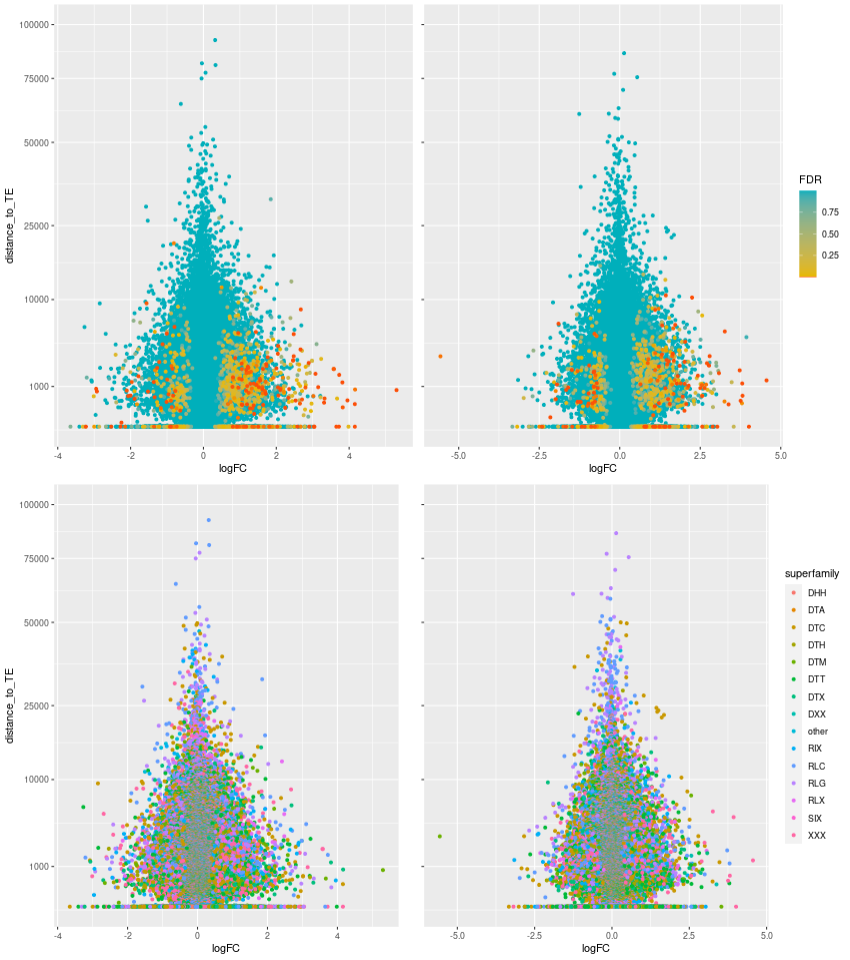
**Supplementary fig. 11** The relationship between up- and down-regulation of genes and their distance to closest TEs in 109xL-C4 (grain). See Supplementary fig. 9 for more details.


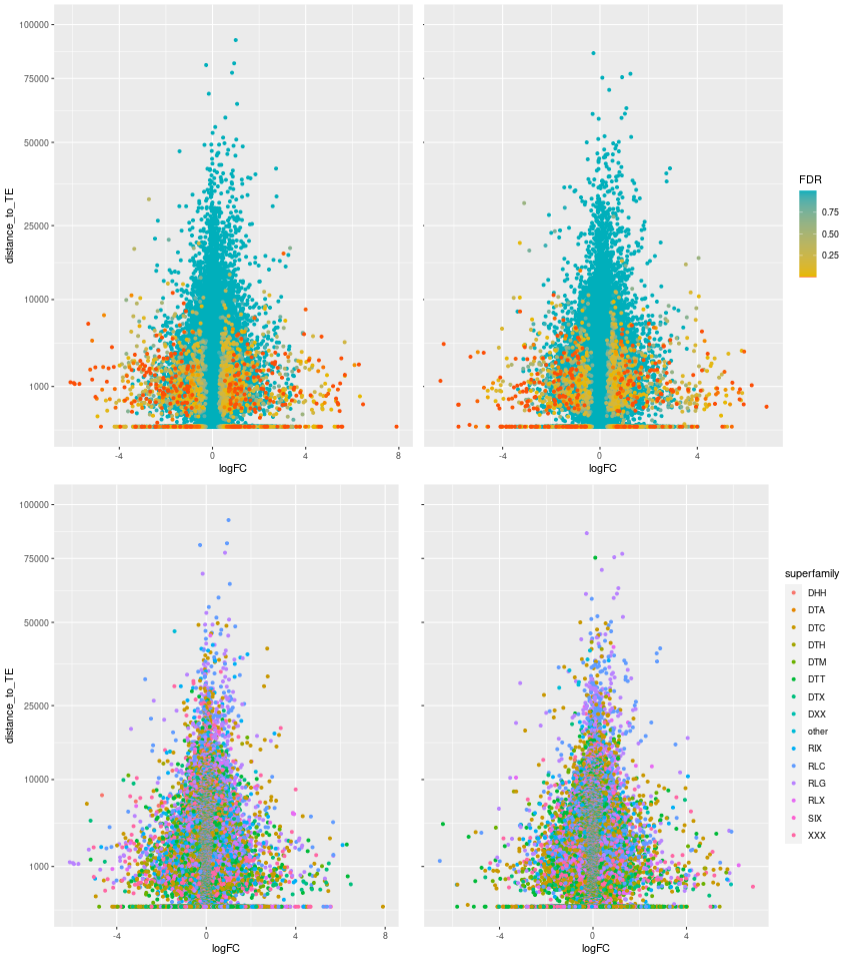
**Supplementary fig. 12** The relationship between up- and down-regulation of genes and their distance to closest TEs in Jx87-S5 (grain). See Supplementary fig. 9 for more details.


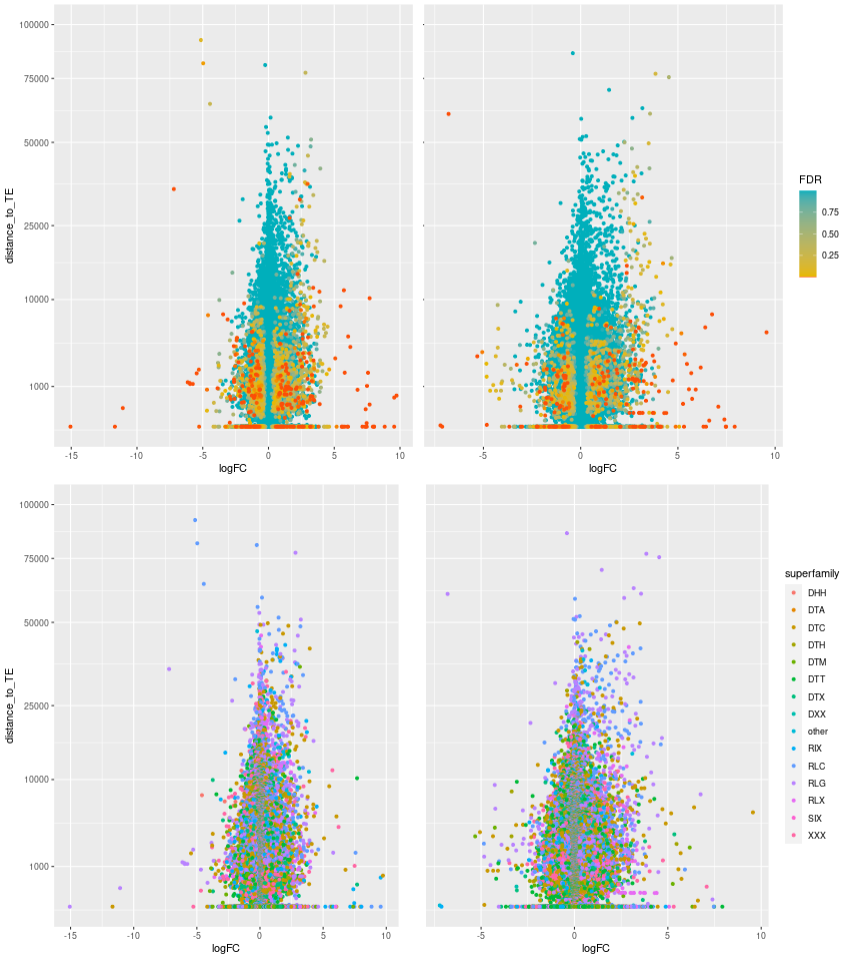
**Supplementary fig. 13** The relationship between up- and down-regulation of genes and their distance to closest TEs in Jx109-S5 (grain). See Supplementary fig. 9 for more details.


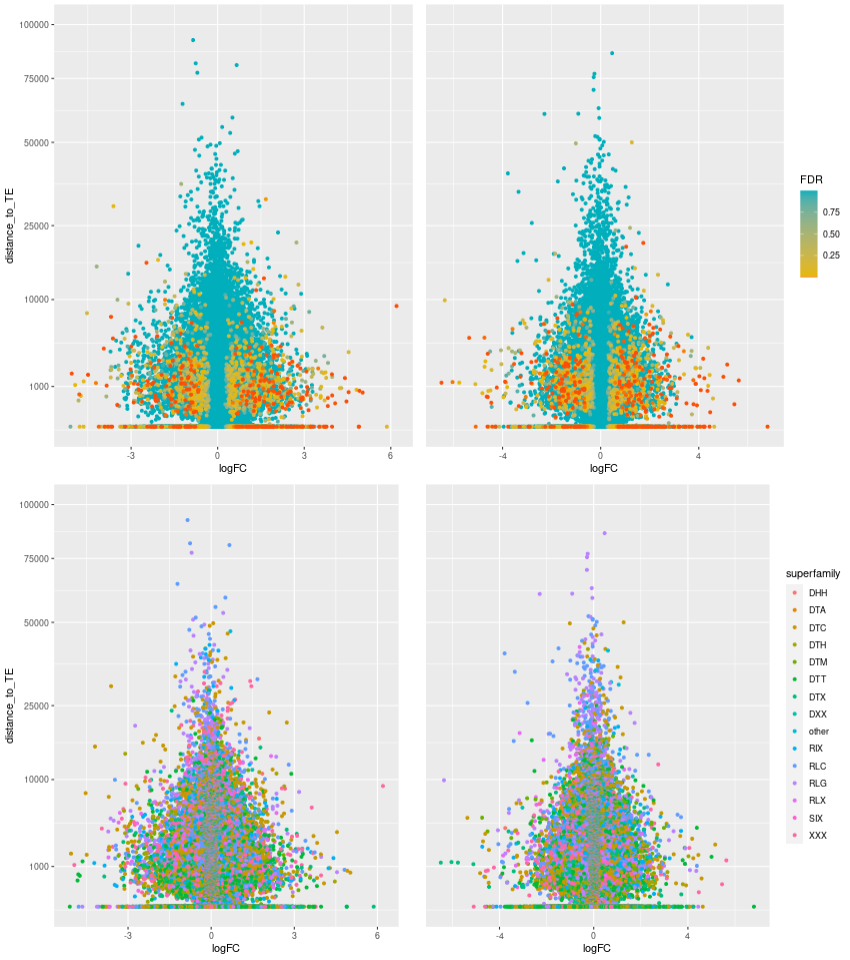
**Supplementary fig. 14** The relationship between up- and down-regulation of genes and their distance to closest TEs in 109xL-C1 (leaves). See Supplementary fig. 9 for more details.


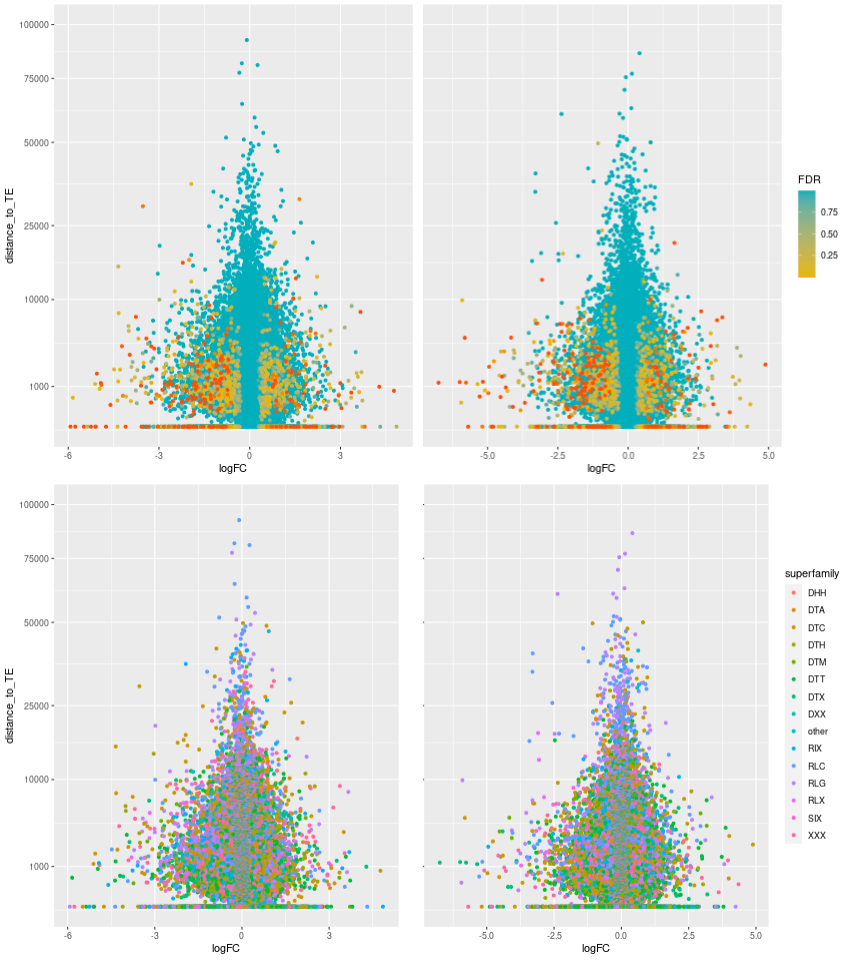
**Supplementary fig. 15** The relationship between up- and down-regulation of genes and their distance to closest TEs in 109xL-C3 (leaves). See Supplementary fig. 9 for more details.


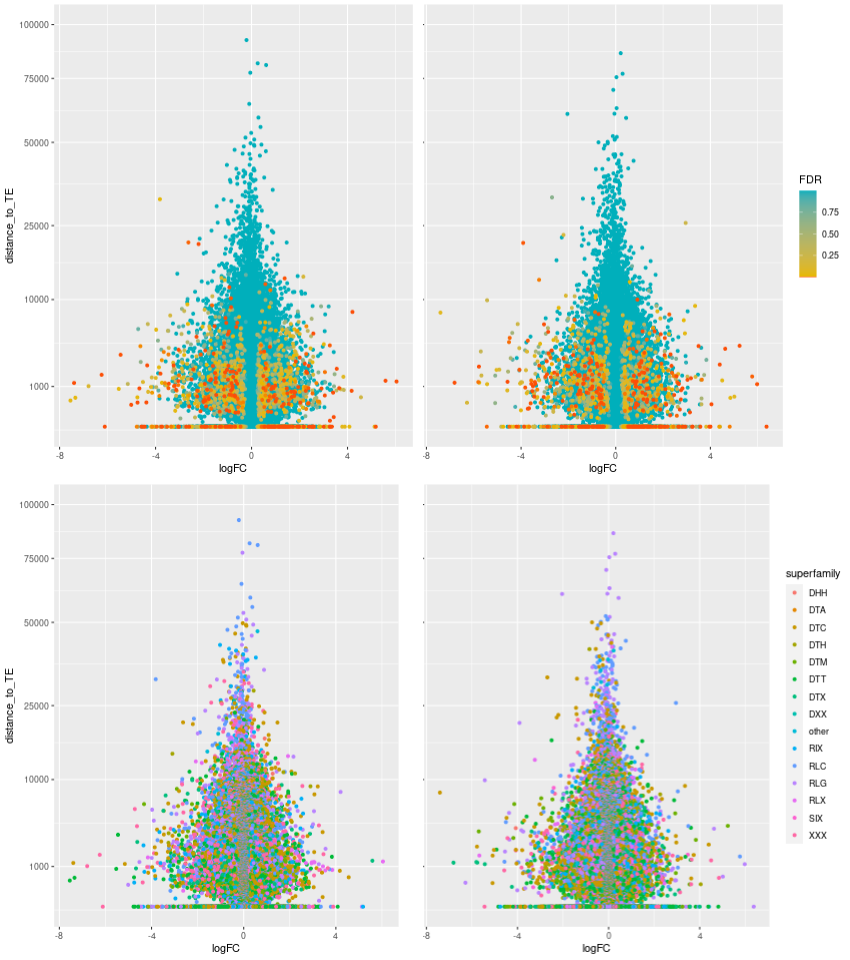
**Supplementary fig. 16** The relationship between up- and down-regulation of genes and their distance to closest TEs in Lx109-C1 (leaves). See Supplementary fig. 9 for more details.


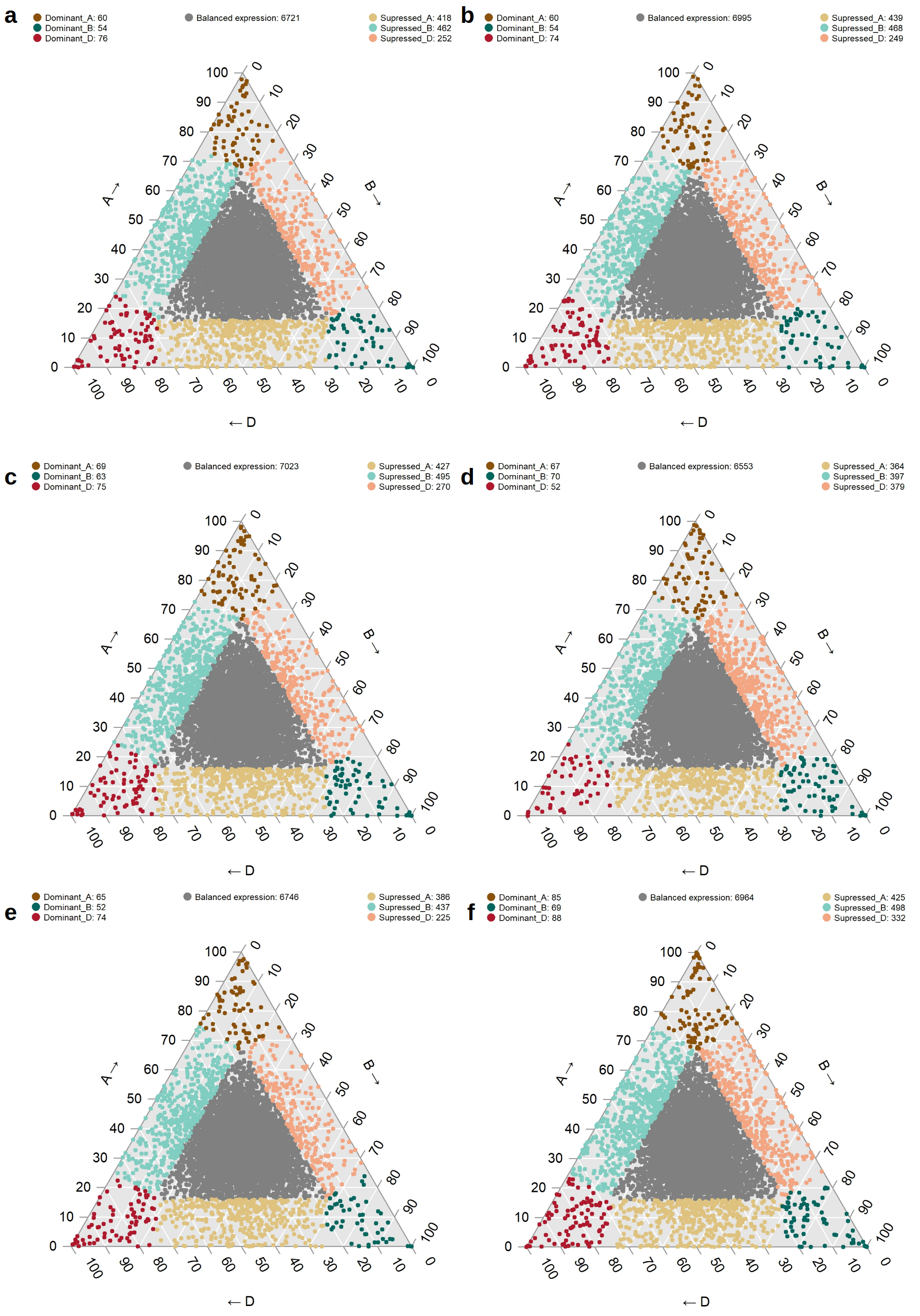


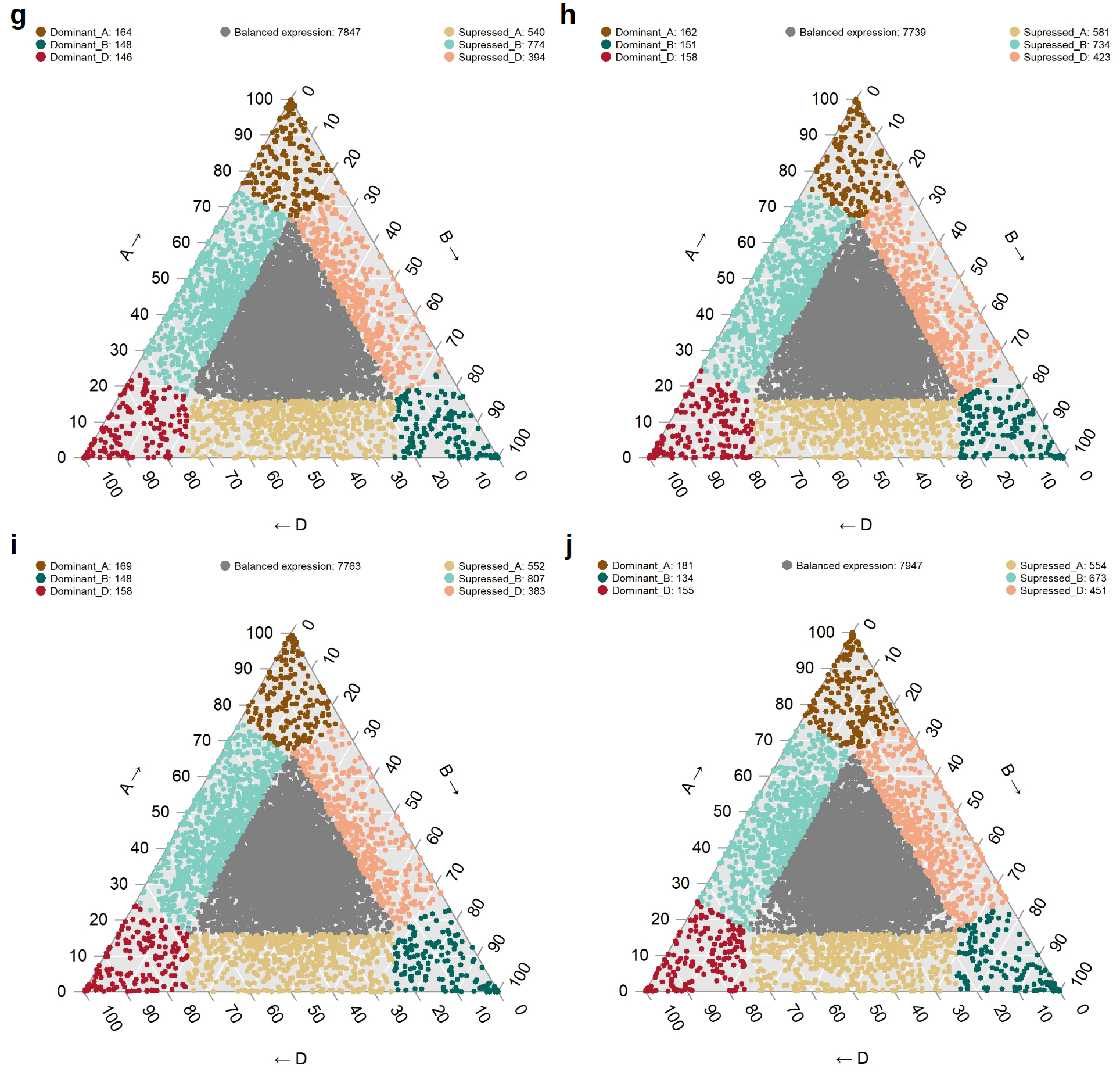


**Supplementary fig. 17 (continued)** Ternary plots showing homoeolog expression bias in the triads of all analysed allohexaploids. Biased triads can be divided into multiple categories, depending whether the triad expression is supplied by a single homoeolog (dominant_A, dominant_B or dominant_D; triads located near the triangle vertices), or by two homoeologs (suppressed_A, suppressed_B and suppressed_D; triads located along the edges in between the vertices). If each of the three homoeologs contributes >0.1667 to its triad expression, the triad is classified as balanced. **a**: 109xL-C2, **b**: 109xL-C4, **c**: Lx109-C2, **d**: Jx87-C2, **e**: Jx109-C2, **f**: Recital (grain), **g**: 109xL-C1, **h**: 109xL-C3, **i**: Lx109-C1, **j**: Recital (leaves).


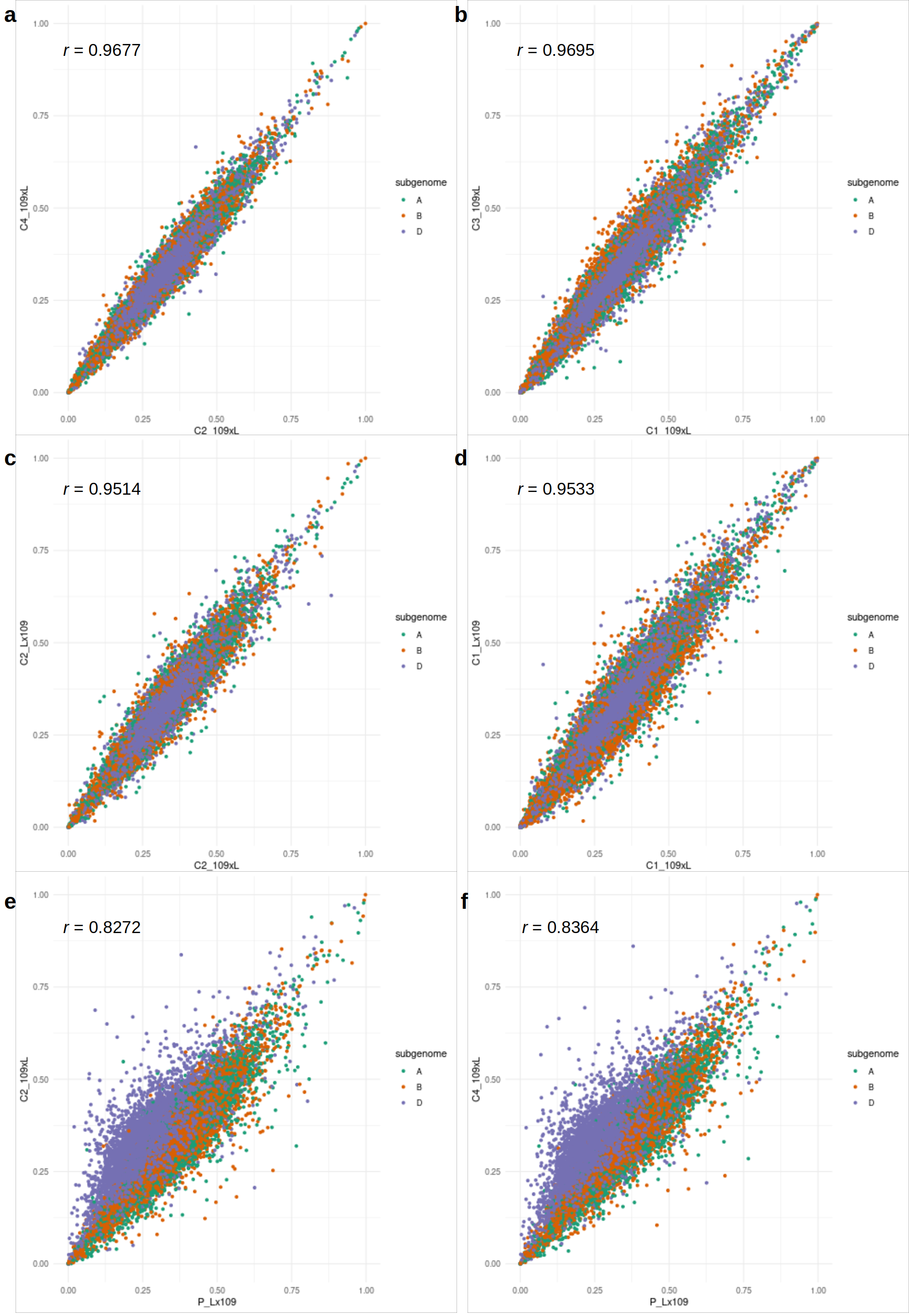


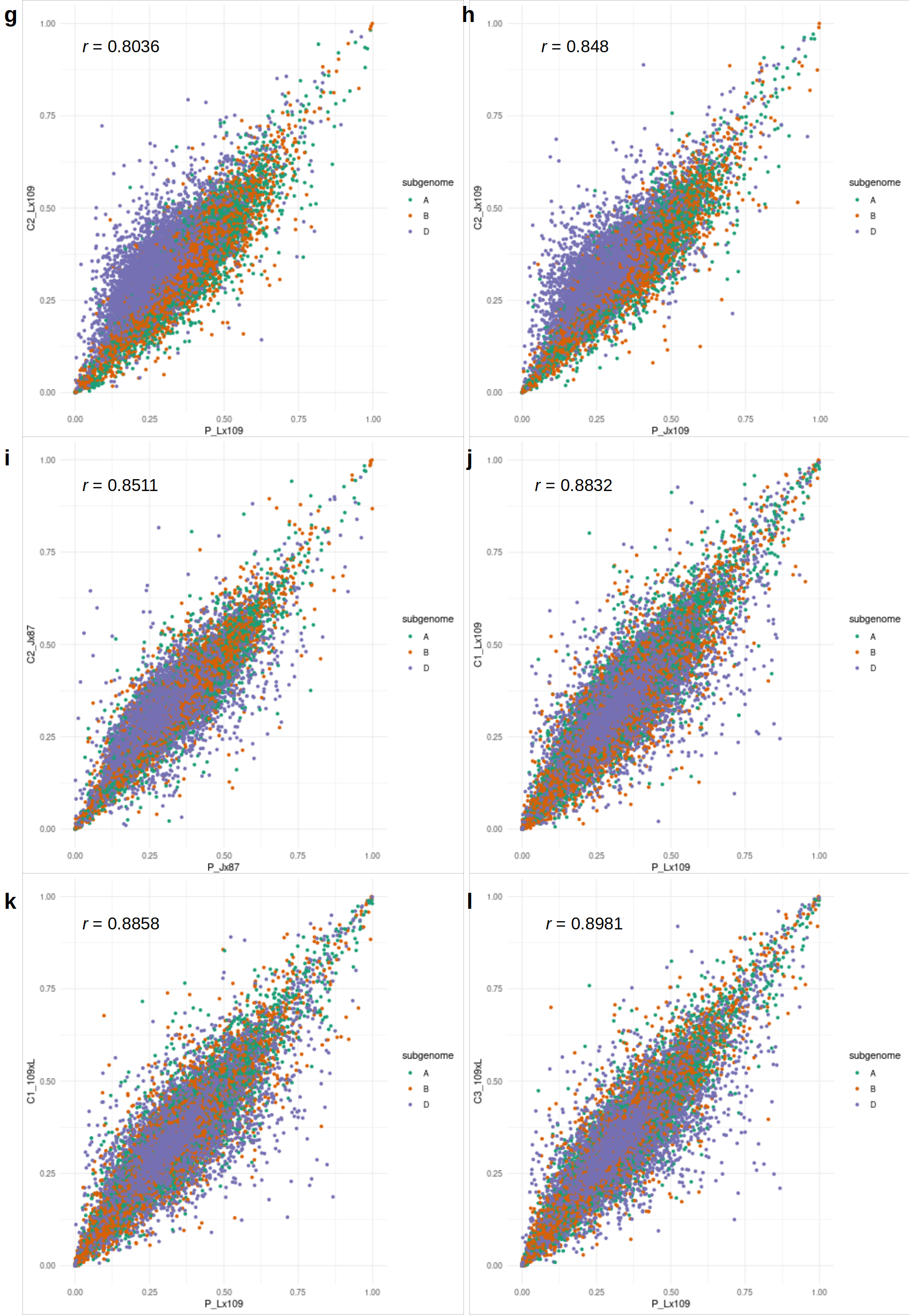


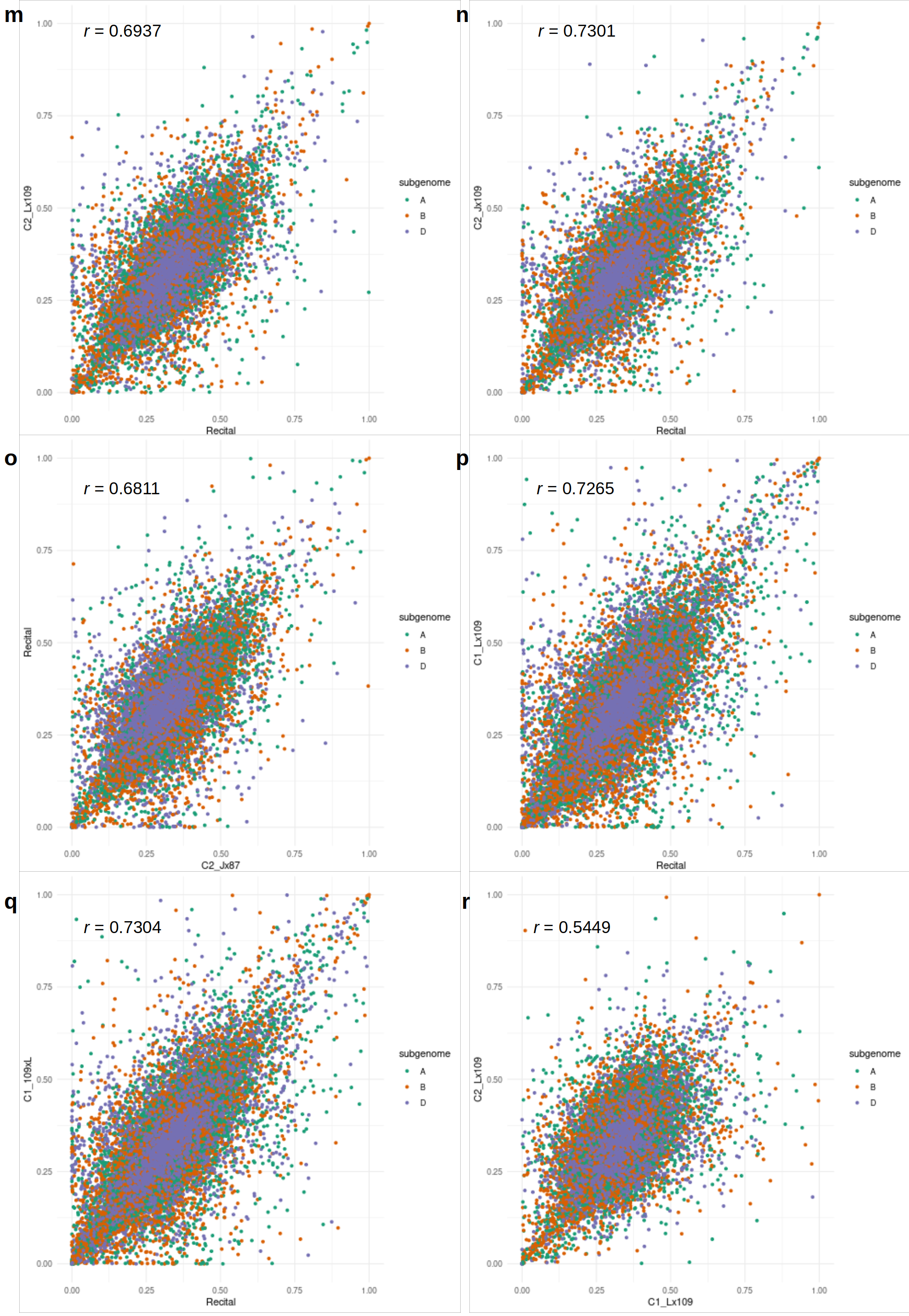


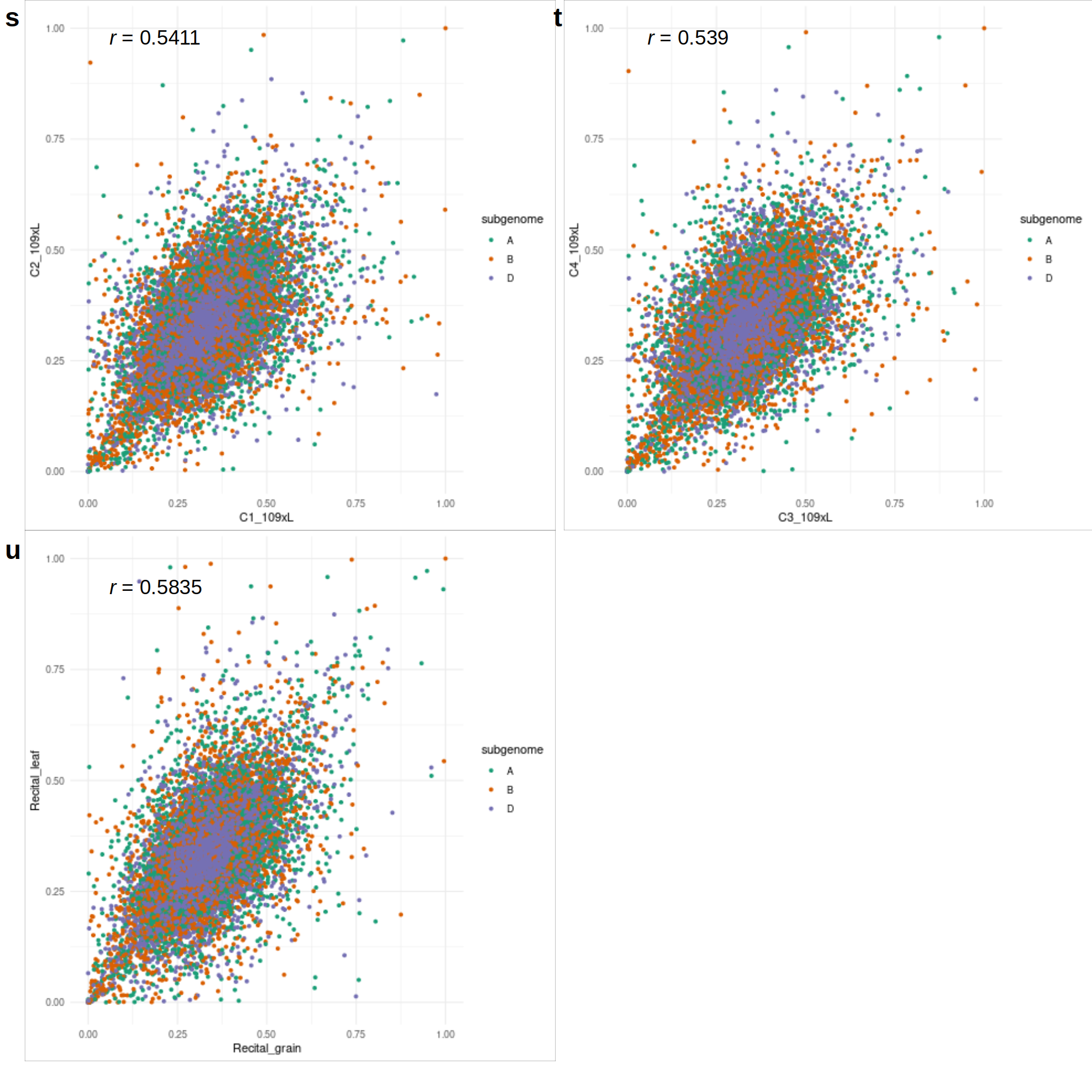


**Supplementary fig. 18 (continued)** Pairwise comparisons of homoeolog expression bias. **a**: 109xL-C2 vs. 109xL-C4 (across generations), **b**: 109xL-C1 vs. 109xL-C3 (across generations), **c**: 109xL-C2 vs. Lx109-C2 (reciprocal crosses), **d**: 109xL-C1 vs. Lx109-C1 (reciprocal crosses), **e**: 109xL-C2 vs. combined parents, **f**:109xL-C4 vs. combined parents, **g**: Lx109-C2 vs. combined parents, **h**: Jx109-S5 vs. combined parents, **i**: Jx87-S5 vs. combined parents, **j**: Lx109-C1 vs. combined parents, **k**: 109xL-C1 vs. combined parents, **l**: 109xL-C3 vs. combined parents, **m**: Lx109-C2 vs. Recital (grain), **n**: Jx109-S5 vs. Recital (grain), **o**: Jx87-S5 vs. Recital (grain), **p**: Lx109-C1 vs. Recital (leaf), **q**:109xL-C1 vs. Recital (leaf), **r**: Lx109-C1 vs. Lx109-C2 (across tissues), **s**: 109xL-C1 vs. 109xL-C2 (across tissues), **t**: 109xL-C3 vs. 109xL-C4 (across tissues), **u**: Recital (grain) vs. Recital (leaf) (across tissues).

**
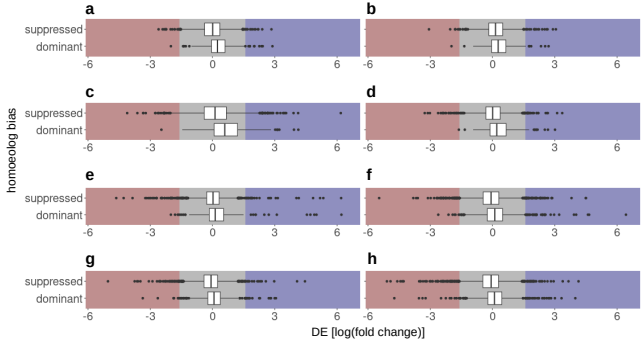
Supplementary fig. 19** Distribution of expression changes (synthetics vs. parents) in dominant and suppressed homoeologs (contribution to triad expression >0.6667 and <0.1667, respectively). Red and blue backgrounds indicate down- and up-regulation DEG zones, respectively, i.e. expression changes below -1.585 and above 1.585 log(fold change) threshold (not considering FDR). The vast majority of dominant and suppressed homoeologs are not differentially expressed (grey background). **a**: 109xL-C2, **b**: 109xL-C4, **c**: Lx109-C2, **d**: Jx109-S5, **e**: Jx87-S5, **f**: 109xL-C1, **g**: 109xL-C3, **h**: Lx109-C1.


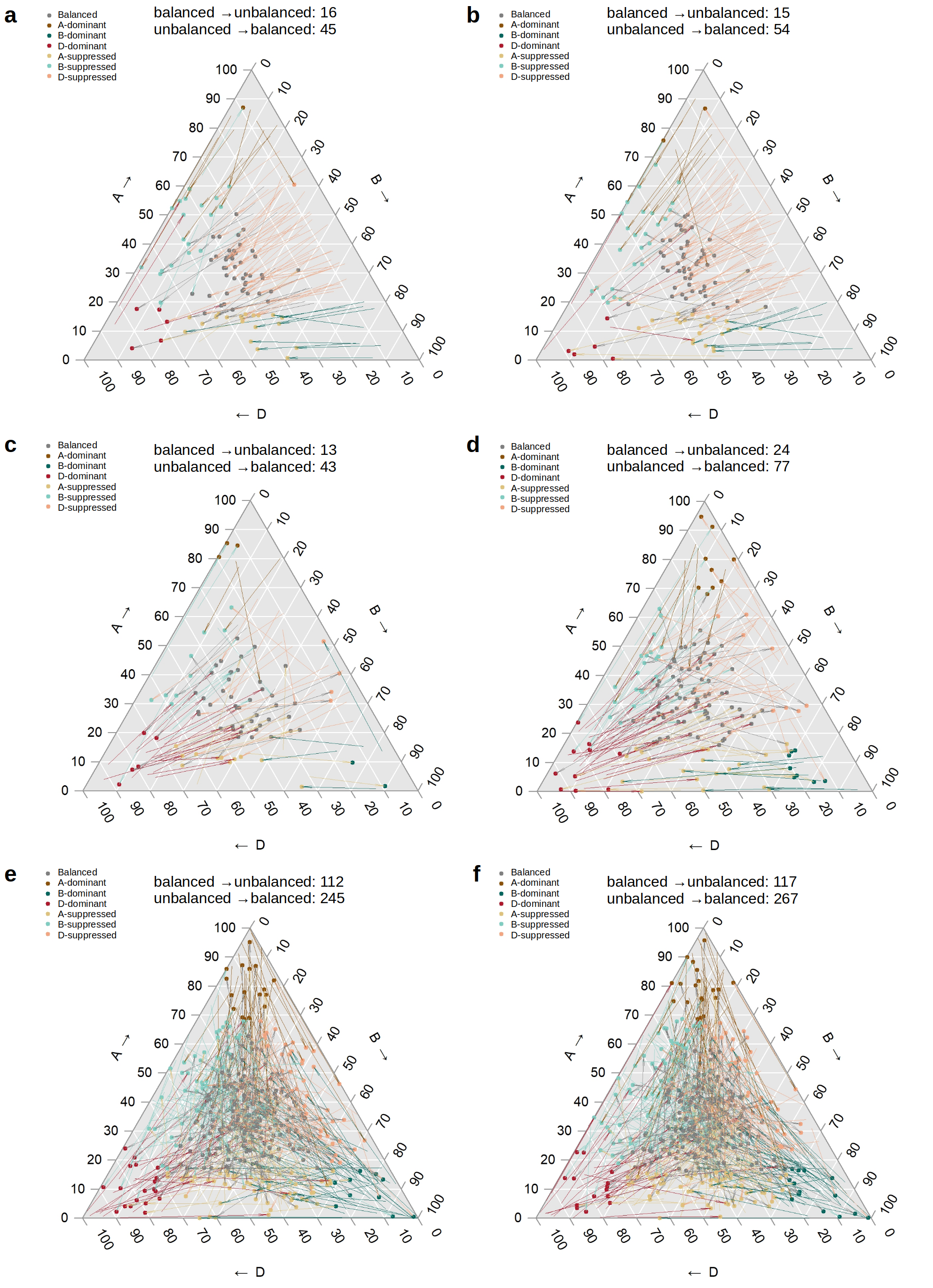


**Supplementary fig. 20** Ternary plots showing triads that changed their position (above a 0.3 eigen distance cut-off) as a result of polyploidization and tissue differentiation. The coloured dots show the triad position in a synthetic, and the arrows indicate its original position in the parents. Grey-coloured arrows indicate that the original triad was balanced, while grey-coloured dots indicate that the new triad position is balanced. Changes that do not involve unbalanced positions, and triads that became activated/inactivated in the synthetic are not shown. **a**: 109xL-C2 vs. combined parents (grain), **b**: Jx109-S5 vs. combined parents (grain), **c**: J87-S5 vs. combined parents (grain), **d**: Lx109-C1 vs. combined parents (leaves), **e**: 109xL-C1 vs. 109xL-C2 (changes across tissues), **f**: 109xL-C3 vs. 109xL-C4 (changes across tissues).
